# Multifaceted antimicrobial mechanisms of NCR147-derived peptides from *Medicago truncatula*

**DOI:** 10.3389/fmicb.2025.1720738

**Published:** 2026-01-27

**Authors:** Hilda Tiricz, Rui M. Lima, Ilona Pfeiffer, Nóra Igaz, Ildikó Domonkos, Sándor Jenei, Dian H. O. Howan, Alexandra Pál, Edit Tímár, Éva Hunyadi-Gulyás, Gábor K. Tóth, Zoltán Bozsó, Éva Kondorosi

**Affiliations:** 1 Institute of Plant Biology, HUN-REN Biological Research Centre, Szeged, Hungary; 2 Department of Biotechnology and Microbiology, Faculty of Science and Informatics, University of Szeged, Szeged, Hungary; 3 Department of Biochemistry and Molecular Biology, University of Szeged, Szeged, Hungary; 4 Department of Medical Chemistry, Albert Szent-Györgyi Medical School, University of Szeged, Szeged, Hungary; 5 Doctoral School of Biology, University of Szeged, Szeged, Hungary; 6 Proteomics Research Group, Core Facility, HUN-REN Biological Research Centre, Szeged, Hungary; 7 MTA-SZTE Biomimetic Systems Research Group, University of Szeged, Szeged, Hungary; 8 Plant Protection Institute, HUN-REN Centre for Agricultural Research, Budapest, Hungary; 9 Centre for Translational Medicine, Semmelweis University, Budapest, Hungary

**Keywords:** antimicrobial activity, bacterial targets, biofilm inhibition, *Candida albicans*, *Cryptococcus neoformans*, efflux pump inhibition, ESKAPE bacteria

## Abstract

**Introduction:**

Antimicrobial peptides (AMPs), key components of innate immunity, offer broad-spectrum activity against diverse pathogens. In *Medicago truncatula*, over 700 nodule-specific cysteine-rich (NCR) peptides with highly diverse sequences and physicochemical properties are produced in the symbiotic cells of root nodules, where cationic members block bacterial cell division and display potent antimicrobial activity *in vitro*. In contrast, anionic NCRs typically lack antimicrobial effects, and NCR147—a neutral peptide—is the only known non-cationic NCR that shows weak bactericidal activity. This unique property prompted us to identify the antimicrobial region of NCR147 and enhance its activity through targeted sequence modifications.

**Materials and methods:**

In this study, 13 truncated and substituted derivatives of NCR147 were chemically synthesized to identify peptide regions responsible for antimicrobial activity. Antimicrobial efficacy was evaluated against 18 pathogens by determining minimum bactericidal and minimum fungicidal concentrations. Inhibition and eradication of bacterial biofilms were assessed to determine peptide effects. Cytotoxicity was measured using hemolysis assays and multiple viability assays in human cell cultures. Peptide interactions with membrane lipids, effects on membrane permeability, and modulation of bacterial efflux pumps were analyzed using established biochemical and biophysical assays. Bacterial proteins interacting with selected peptides were identified by affinity chromatography followed by LC–MS/MS.

**Results:**

The NCR147 derivatives displayed varying degrees of antimicrobial potency and spectrum. Analysis of the physicochemical properties and predicted 3D structures of 13 NCR peptide variants revealed that the antimicrobial region resides in the C-terminal portion of these intrinsically disordered peptides, where the WAW hydrophobic patch together with the positively charged amino acids contribute to antimicrobial activity, most likely through interactions with microbial membranes. The most active peptides provoked alteration of bacterial membranes, inhibited efflux pumps, and interfered with essential intracellular targets. Moreover, these peptides exhibited potent antibiofilm effects, including the ability to both prevent and degrade *Acinetobacter baumannii* biofilms. Incorporation of 5-fluoro-L-tryptophan enhanced both antimicrobial breadth and antifungal activity. Importantly, this fluorinated peptide was non-cytotoxic to human cells.

**Discussion:**

These findings reveal that NCR147-derived peptides function via a multihit mechanism and highlight the therapeutic promise of plant-derived AMPs as next-generation antimicrobials with reduced risk of resistance development.

## 1 Introduction

Antibiotic resistance has been steadily increasing for decades across the globe, posing a growing challenge and burden on both the food supply and the healthcare sector in our overpopulated world. Various organisms, including humans, animals and plants, produce a class of defense molecules known as antimicrobial peptides (AMPs). These peptides play a crucial role in the innate immune response against a wide range of infections. AMPs exhibit a broad spectrum of activity, effectively capable of eliminating or inhibiting the growth and survival of diverse microorganisms, including bacteria, fungi, viruses, protozoa, and their antimicrobial mechanisms are different from traditional antibiotics (Mookherjee et al., 2020). This significance becomes particularly pronounced when considering the serious global health challenge of antibiotic resistance (Hancock, 1997; Hancock and Sahl, 2006). In the Collection of Anti-Microbial Peptides (CAMP), there are currently 11,827 natural and 12,416 known synthetic AMPs (Gawde et al., 2023). Despite their variable physicochemical properties, they predominantly possess cationic features. Their positive charge enables them to adhere to the negatively charged membranes of both Gram-positive and Gram-negative bacteria and their antimicrobial properties generally based on destabilization and damage of microbial cell membranes.

In plants, defensins, characterized by the presence of 8 cysteine residues forming 4 disulfide bridges, represent the largest class of AMPs (Parisi et al., 2019). More than 1,200 plant defensins are known and among the few tested ones the majority exhibit antifungal activities though a few also have antibacterial activities, while they are non-toxic to mammalian and plant cells (Ishaq et al., 2019). The large sequence variations of defensins can confer different functions, including binding to specific lipids, inhibiting protein synthesis, interacting with nucleic acids, and inhibiting ion channels (Carvalho and Gomes, 2009).

Leguminous plants with their Rhizobium soil bacterium partners form root nodules where bacteria are converted to nitrogen fixing bacteroids. In the nodules of *Medicago truncatula* and related species, the bacteria undergo a multistep, plant directed irreversible terminal differentiation process resulting in enlarged polyploid uncultivable bacteroids with altered morphology and increased membrane permeability (Mergaert et al., 2006; Montiel et al., 2017). The plant effectors of terminal bacteroid differentiation are defensin-related Nodule-specific Cysteine-Rich NCR peptides (Van De Vel et al., 2010). In *M. truncatula*, over 700 NCR peptides participate in this intricate process involving different sets of NCRs in the early, middle and late stages of symbiotic cell development (Mergaert et al., 2003, 2006; Van De Vel et al., 2010; Kondorosi et al., 2013; Montiel et al., 2017). The NCR genes have exclusively evolved in legumes with terminal differentiation of bacteroids and are only expressed in the symbiotic cells (Van De Vel et al., 2010; Kondorosi et al., 2013). The NCR peptides are encoded by small genes and built up by a relatively conserved signal peptide and a mature peptide containing 4 or 6 cysteine residues in conserved positions in a typically 24–50 amino acids long, highly variable sequence with diverse physicochemical properties (Lima et al., 2020). The cysteine residues allow the formation of 2 or 3 disulfide bridges, but in *in vitro* experiments NCRs are also active in their reduced state with disordered structure. NCRs are specifically targeted to the endosymbiotic Rhizobium bacteria (called bacteroids) and one of their key functions is irreversible inhibition of bacterial cell division.

Multiple studies have demonstrated that cationic NCR peptides possess antimicrobial and antifungal properties (Tiricz et al., 2013; Balogh et al., 2014; Ördögh et al., 2014; Nagy et al., 2015; Mikuláss et al., 2016; Farkas et al., 2017; Jenei et al., 2020; Velivelli et al., 2020; Isozumi et al., 2021; Szerencsés et al., 2021, 2025) affecting membrane integrity and biological processes by interacting with various proteins (Lima et al., 2020; Isozumi et al., 2021). In the most comprehensive study so far 78 NCR peptides from *M. truncatula* were tested against eight pathogens including the ESKAPE pathogens: *Enterococcus faecalis, Staphylococcus aureus*, *Klebsiella pneumoniae*, *Acinetobacter baumannii*, *Pseudomonas aeruginosa* and *Enterobacteriaceae* including *Escherichia coli* and *Salmonella enterica*, and 26 of them showed antimicrobial activity (Lima et al., 2022). Three of them killed all tested pathogens, while the others were effective against several or at least one of these pathogens at micromolar concentrations. Among the 26 peptides, 25 shared the common feature of being cationic and having a positive net charge. However, while these features were required, they did not necessarily guarantee antimicrobial activity as it was also influenced by the amino acid sequence. The only non-cationic peptide with a modest antimicrobial activity against *A. baumannii*, *Listeria monocytogenes*, *E. coli* and *P. aeruginosa* was a 36 amino acid long neutral peptide, NCR147 with isoelectric point (pI) 7.61 and net charge 1. Unlike the cationic AMPs, which interact with the negatively charged microbial membranes, it was unknown how this neutral peptide exerts its antimicrobial activity: whether the full mature peptide sequence is required for the antimicrobial activity or whether a shorter sequence is sufficient. In this study, we aimed to identify the peptide region responsible for the antimicrobial activity of NCR147. To achieve this, various regions of NCR147 were synthesized, mutations were introduced, and modified amino acids were incorporated into the sequence. The antimicrobial activity of NCR147-derived peptides was assessed against 16 bacterial species, including both human and plant pathogens. In addition to bactericidal effects, we evaluated their ability to inhibit and eradicate biofilms, disrupt bacterial membranes, interact with membrane lipids, inhibit efflux pumps, and bind key bacterial proteins—collectively indicating a multifaceted mechanism of action. Substitution with 5-fluoro-L-tryptophan significantly enhanced the antimicrobial properties, extending the killing effect to fungal pathogens such as *Candida albicans* and *Cryptococcus neoformans* without exhibiting cytotoxicity to normal human cells.

## 2 Materials and methods

### 2.1 Bacterial strains

The human pathogenic bacterial strains obtained from the ATCC (United States) and NCTC (National Collection of Type Cultures – England) were *Enterococcus faecalis* (ATCC 29212), *Staphylococcus aureus* (HNCMO112011), *Listeria monocytogenes* (ATCC 19111), *Pseudomonas aeruginosa* (ATCC 27853), *Escherichia coli* (ATCC 8739), *Salmonella enterica* (ATCC 13076), *Klebsiella pneumoniae* (NCTC 13440), *Acinetobacter baumannii* (ATCC 17978). *Pseudomonas syringae* pv. *tomato* DC3000 (Cuppels, 1986), *Pseudomonas syringae* pv. *tabaci* (B.01601)*, Pseudomonas syringae* pv. *maculicola Lux CDABE* (Fan et al., 2008), *Burkholderia gladioli* previously “*Pseudomonas gladioli* pv. *alliicola”* (B.01675), *Xanthomonas campestris* pv. *malvacearum* previously “*Xanthomonas citri* pv. *malvacearum”* (B.01712), *Dickeya chrysanthemi* previously *“Erwinia chrysanthemi”* (B.01392), *Pectobacterium atrosepticum* previously “*Erwinia carotovora* ssp. *atroseptica”* (B.01611) were obtained from National Collection of Agricultural and Industrial Microorganisms, Budapest Hungary. As *Agrobacterium tumefaciens* the C58C1 (Deblaere et al., 1985) strain was used.

### 2.2 Chemical synthesis of peptides

Peptides were synthesized according to the standard procedure of the solid-phase peptide synthesis (SPPS) by using an automatic peptide synthesizer (CEM Liberty Blue, United States, NC) (the loading of the amino groups was 0.23 mmol/g). The applied chemistry utilized the Fmoc amino protecting group and diisopropylcarbodiimide/oxyma coupling with a fivefold excess of reagents. Removal of the fluorenyl-9-methoxycarbonyl group was carried out with 10% piperazine and 0.1 mol 1-hydroxybenzotriazole dissolved in 10% ethanol and 90% DMF in 2 cycles. After completion of the synthesis, the peptides were detached from the resin with a 95:5 (v/v) trifluoroacetic acid (TFA)/water mixture containing 3% (w/v) dithiothreitol and 3% (w/v) triisopropylsilane at room temperature for 3 h. The resin was removed by filtration, and the peptides were precipitated by the addition of ice-cold diethyl ether. Next, the precipitate was filtered, dissolved in water and lyophilized. The crude peptides were analyzed and purified by reverse-phase high-performance liquid chromatography (RP-HPLC). Peptides were purified using a C18 column with a solvent system of (A) 0.1% (v/v) TFA in water and (B) 80% (v/v) acetonitrile and 0.1% TFA (v/v) in water at a flow rate of 4.0 mL min^−1^. The absorbance was detected at 220 nm. The appropriate fractions were pooled and lyophilized. Purity of the final products were characterized by analytical RP-HPLC at a flow rate of 1.0 mL min^−1^. The identity of the peptides was proved by electrospray ionization mass spectra using Waters SQ detector. The measured m/z values were in good agreement with the calculated ones. The purity of the peptides used was above 95% in all cases (Supplementary Figure S1).

### 2.3 Antimicrobial activity in PPB

Human pathogenic bacteria were grown at 37 °C, while plant pathogens at 30 °C in liquid LB medium (10 g/L tryptone, 5 g/L yeast extract and 10 g/L sodium chloride). Overnight starter cultures were diluted hundred-fold and grown to OD_600_ = 0.2–0.6. After harvesting and washing bacteria with 20 mM Potassium-Phosphate Buffer, pH 7.4 (PPB), the suspensions were diluted in PPB to OD_600_ = 0.1 corresponding to ∼10^7^ bacteria/mL. Antimicrobial activity of the peptides and the antibiotics was determined in 96 well microplates using 10 μL OD_600_ = 0.1 bacterial suspension (OD_600_ = 0.01 at final concentration) and 90 μL of the 2-fold serial dilutions of peptides or antibiotics in PPB. Peptides were tested from 100 or 50 μM to 0.2 μM. The designed peptide derivatives were chemically synthesized as described above, and the antibiotics were purchased from Sigma Aldrich, St. Louis, MO, United States: levofloxacin (Prod. No. 28266-10MG-F), cefepime hydrochloride (cefepime) (Prod. No. A3737-10MG), meropenem trihydrate (meropenem) (Prod. No. 119478–56-7). The microplates were incubated for 3 h at 30 °C/37 °C with 250 rpm and then 5 μL from each sample were placed on LB agar plates to monitor the growth of bacteria. The lowest concentration of the antimicrobial agents, which eliminated viable bacteria was the minimum bactericidal concentration (MBC). All measurements were repeated at least three times.

### 2.4 Antimicrobial activity of *Acinetobacter baumannii* in Mueller-Hinton broth

Starter and log phase cultures of *A. baumannii* were prepared similarly as described above but the bacteria were resuspended in 1/10 Mueller-Hinton Broth (Beef solids 0.2 g/L, Starch 0.15 g/L, Casein hydrolysate 1.75 g/L with final pH 7.4 at 25 °C). 10 μL of bacteria at OD_600_ = 0.1 supplemented with 90 μL of 2-fold dilution series of peptides or antibiotics in 1/10 MHB were cultivated for 3 h at 37 °C with 250 rpm. Then 5 μL aliquots were placed on the top of LB agar plates and bacterial growth was monitored after overnight incubation at 37 °C. The measurements were repeated 3–5 times.

### 2.5 Biofilm formation, inhibition and eradication assay on *Acinetobacter baumannii*

Biofilm formation in our assays was performed using a standard microtiter-plate biofilm model (O’Toole, 2011) where bacterial cells attach to the bottom and form a ring-shaped biofilm along the air–liquid interface on the side walls of the wells. *A. baumannii* starter culture was grown in LB at 37 °C. Following day, the culture was diluted 100-fold in 1/2 MHB and grown until reaching OD_600_ = 0.4–0.6. This log phase culture was centrifuged with 4,000 rpm and resuspended in the same medium and adjusted to OD_600_ = 1.00.

For biofilm inhibition assay 2 μL of bacterial suspension were treated with two-fold dilution series of NCR147 derivatives in concentrations ranging from 100 μM to 3.13 μM in 200 μL final volume (bacteria OD_600_ = 0.01 at final concentration). Biofilm was grown in 96-well plates for 24-h with slow agitation (50 rpm) at 37 °C. The formed biofilm was then washed three times with 250 μL of sterile water.

For biofilm eradication assay biofilms grown in 96-well microtiter plates at 37 °C for 24-h with slow agitation (50 rpm) were washed three times with 200 μL of 1/10 MHB. The washed biofilms were exposed to NCR147 peptide derivatives (100 μM-3.13 μM) diluted in 1/10 MHB, followed by a 3-h incubation period with gentle shaking for biofilm eradication at room temperature. After the medium was removed and the remaining biofilm was washed three times with water.

The amount of biofilms in both the inhibition and eradication assays was measured by adding 200 μL of 1% Crystal Violet (CV) for 15 min at room temperature. Non-adherent dye was eliminated through triple washings with water and the thoroughly dried sample was dissolved in 200 μL of 30% acetic acid with a 15-min incubation at room temperature. The absorbance of the dissolved dye was measured at OD_550_ using a Hidex Sense Microplate Reader (O’Toole, 2011; Allkja et al., 2021). The measurements were repeated three times. Representative images of biofilm formation, inhibition, and eradication are shown in Supplementary Figure S2.

### 2.6 Luminescent cell viability assay

Bioluminescence of luxCDABE was measured in 100 μL of log phase culture of *P. syringae* pv. *maculicola luxCDABE* (*Psm*) and *P. syringae* pv. *tomato* DC3000 *luxCDABE* (*Pstm*) at OD_600_ = 0.1 in the presence of 20 μM NCR147 peptide derivatives in PPB with readings in every 5 min using a Hidex Sense Microplate Reader for 30 min. The growth of bacteria was tested on agar plates. The measurements were repeated three times.

### 2.7 Membrane lipid-peptide interaction assay

The membrane lipid strips (catalog No. P-6002) purchased from Echelon Bioscience (Salt Lake City, UT, United States) were used according to the recommended protocol. Briefly, the membranes were incubated with NCR147_25-36_-StrepII or NCR147_25-36_ W_33,35_W^F^-StrepII at 10 μg/mL for 1 h and after washing the strips, the binding of Strep II tagged peptides to lipids was detected with StrepTactin-horseradish peroxidase (HRP) conjugate (BioRad Cat. No. 1610380) using chemiluminescence of HRP substrate with ECL Prime Western Blotting Detection Reagent (Cytiva Cat. No. GERPN2236).

### 2.8 Efflux pump assay

An overnight culture of *E. coli* and *A. baumannii* in LB medium at 37 °C were 100-fold diluted and grown until OD_600_ = 0.4–0.6 when the bacteria were harvested with centrifugation, washed three times with PPB and resuspended in PPB to OD_600_ = 0.15 corresponding to approximately 1.2×10^8^ CFU/mL. The 100–100 μL suspension was placed into a 96-well microtiter plate, and ethidium bromide (EtBr) was added to a final concentration of 10 μg/mL. Fluorescence was monitored using a HIDEX plate reader, with excitation at 530 nm and emission at 600 nm, measured every minute for 20 cycles. NCR147 derivatives or antibiotics were added at the second cycle.

### 2.9 Propidium iodide uptake

Propidium iodide (PI) at 10 μg/mL and NCR147_25-36_ or NCR147_25-36_ W_33,35_/W^F^ at 6.25 μM were added simultaneously to log phase (OD_600_ = 0.1) *E. coli* and *A. baumannii*. The presence of intracellular PI bound to nucleic acids was monitored in Hidex Sense Microplate Reader over a 60-min period, with readings in every minute.

### 2.10 Hemolysis assays on human red blood cells

Human blood was purchased from the Regional Blood Center in Szeged. The use of human blood has been authorized by the Regional Hungarian Ethics Committee and approved by the Ethics Review Sector of DG RTD (European Commission) in connection with EK’s ERC AdG SymBiotics. The cells from 1.2 mL of EDTA-blood were centrifuged at 1500 rpm for 1 min and washed several times in TBS buffer (10 mM Tris, pH 7.2, 150 mM NaCl) until the supernatant became colorless. The cells were then resuspended in 12 mL TBS buffer. 100 μL of this cell suspension was incubated with peptides at 100 μM final concentration for 1 h at 37 °C. After centrifugation of the cells at 1500 rpm for 1 min the supernatants were transferred into sterile 96-well plates, and the hemoglobin release was measured at OD_560_ in the Hidex Sense Microplate Reader. Triton X-100 (Merck/Order Number:108603.1000) at 100 μM and TBS without peptides were used as positive and negative controls, respectively. Relative hemolytic activity (RHA) of each peptide was calculated as follows: RHA = {(OD_Compound_ − OD_TBS_) × 100} / (OD_Triton X-100_ − OD_TBS_). The measurements were repeated three times.

### 2.11 Affinity chromatography assay

Crude protein extract was prepared from logarithmic phase *E. coli* (OD_600_ = 0.4) by sonication and removing the debris with centrifugation at 15000 rpm. The NCR147_25-36_-StrepII, NCR147_25-36_ W_33,35_W^F^-StrepII or StrepII (as a control) was added at 0.02 μM final concentration to supernatant and incubated on streptavidin resin for 120 min with gentle movement on ice. Affinity chromatography was carried out on Strep-Tactin Sepharose beads (IBA Lifesciences, Göttingen, Germany, Cat. No. 2–1,201-010) according to the manual. Unbound proteins were removed by washing the column seven times with Washing Buffer (100 mM Tris–HCl pH 8.0, 150 mM NaCl, 1 mM EDTA). Proteins bound to NCR147_25-36_-StrepII, NCR147_25-36_ W_33,35_W^F^-StrepII or StrepII were eluted with Elution Buffer (100 mM Tris–HCl pH 8.0, 150 mM NaCl, 1 mM EDTA, 2.5 mM desthiobiotin) and separated by electrophoresis on 4–20% precast denaturing gel (Bio-Rad Laboratories, Hercules, Ca, United States, Cat. No. 456–1,093) and visualized by silver staining. Proteins were identified from the elution fractions with the LC–MS/MS described in 2.12. The mass spectrometry proteomics data have been deposited to the ProteomeXchange Consortium via the PRIDE (Perez-Riverol et al., 2024) partner repository with the dataset identifier PXD060397.

### 2.12 Liquid chromatography mass spectrometry analysis

Protein content of the eluates was digested with trypsin using the S-Trap micro spin columns according to the vendor’s protocol.^1^ Briefly: Samples were diluted twofold with 10% SDS, 100 mM TEAB pH 8.5 and sonicated for 5 min. Proteins were reduced with 5 mM TCEP in 55 °C for 15 min and alkylated with 20 mM MMTS in RT for 10 min. The pH was decreased below 1 with the addition of phosphoric acid and proteins were trapped on the S - Trap™ micro spin column. After washing, 1 μg of Trypsin (Pierce Trypsin Protease, MS grade) was added and incubated at 47 °C for 2 h. Tryptic peptides were eluted and dried before LC–MS/MS analysis using an Evosep One system (Evosep Biosystems) on-line coupled to an FAIMS-Orbitrap Fusion Lumos Tribrid (Thermo Scientific) mass spectrometer. The 15-samples-per-day method with its preprogrammed gradient was applied using a performance column (EV-1137 column, 15 cm x 150 μm, 1.5 μm), the flow rate was 220 nL/min. Peptides eluted from the column were analyzed in 3-s cycles selecting the most abundant multiply charged ions (z = 2–6, m/z range: 300–1,500) for HCD fragmentation (normalized collision energy: 35%) following each MS1 scan. Ion mobility separation was applied with two compensation voltages (−50 and −70). Both MS and MS/MS spectra were collected in the Orbitrap analyzer with a resolution of 120,000 or 50,000, respectively.

Database research was done using Proteome Discoverer (v3.0 SP1, Thermo Scientific) software. Proteins were identified using the ProteinProspector (ver.: 6.5.0) search engine with the following parameters: database: UniProtKB. *Escherichia coli* sequences (2022.07.20. version, 13,227,860 sequences) concatenated with a reversed version for each entry; enzyme: trypsin allowing one missed cleavage site; modifications: static: methylthio on Cys; dynamic: oxidation of Met, acetylation on protein N-term and pyroGlutamine formation on Peptide N-term Gln, allowing up to 2 variable modifications/peptide; mass accuracy: 5 ppm and 25 ppm for precursor and fragment ions, respectively. Peptide level false discovery rate was below 1% for all samples as estimated by the incidence of reversed sequence identifications.

### 2.13 Fungal strains and cultivation conditions

*Candida albicans* SC 5314 (SC: Squibb Institute for Medical Research, New Brunswick, New Jersey, United States) and *Cryptococcus neoformans* IFM 5844 (IFM: Culture Collection of the Research Center for Pathogenic Fungi and Microbial Toxicoses, Chiba University, Chiba, Japan) strains were cultivated for 14 h in YPD medium (1% pepton, 1% dextrose, 0.5% yeast extract) at 30 °C in water bath shaker. The cells were harvested by centrifugation (10 min, 5,000 g) washed twice in sterile distilled water and suspended in YNB medium (five-fold diluted Difco Yeast Nitrogen Base w/o Amino Acids medium (Becton, Dickinson and Company, Sparks, MD, United States) supplemented with 1% dextrose). The cell number was determined in Bürker chamber and adjusted to 5×10^4^ cells/mL concentration.

### 2.14 Detection of the antifungal activity

The antifungal activity of NCR147_25-36_ W_33,35_/W^F^ was tested on in 96-well microtiter plates. The minimum inhibitory concentration (MIC) was established by the micro-dilution method. Briefly, 5 μL serially two-fold-diluted peptides from 25 μM to 1.56 μM were added to 95 μL of cell suspension (5×10^4^ cell/mL) in YNB medium and the plates were incubated for 48 h at 30 °C. The optical density of the cultures was measured at 620 nm in Synergy HTX plate reader (BioTek, Biotek Instruments lnc, Winooski, VT, USA). Minimum inhibitory concentration was defined as growth inhibition ≥ 90% compared to 100% growth of the untreated control.

To establish the fungicidal activity of the peptide, both control and peptide-treated cultures from the MIC assays were diluted 10-, 100- and 1,000-fold in sterile distilled water. Five μL from each dilution was inoculated onto solid YPD medium (1% pepton, 1% dextrose, 0.5% yeast extract, 2% agar). The plates were incubated at 30 °C for 48 h. Minimum fungicidal activity (MFC) was defined as the lowest peptide concentration at which no visible fungal growth was detected. The experiments were carried out four times with two or three replicates.

### 2.15 Human cell cultures

The MRC-5 human fibroblast non-cancerous cell line (ATCC CCL-171) was cultured in Dulbecco’s Modified Eagle Medium (DMEM, Biosera, Cholet, France) containing 1 g/L glucose, 10% FBS (Biosera, Cholet, France), 2 mM glutamine (Biosera, Cholet, France), 0.01% streptomycin, and 0.005% penicillin (Biosera, Cholet, France). This cell type was maintained under standard conditions at 37 °C, with 5% CO_2_ and 95% humidity.

### 2.16 MTT assay

The effect of NCR147_25-36_ W_33,35_/W^F^ peptide on cell viability was assessed using MTT assay. MRC-5 cells were seeded in 96-well plates at a density of 10^4^ cells/well and left to grow. On the following day, the cells were washed with PBS and were treated with 0, 6.25, 12.5, and 25 μM of NCR147_25-36_ W_33,35_/W^F^ peptide diluted in serum-free DMEM media for 24 h. As a positive control, 5 μM cisplatin treatment was performed. After 24 h, the solutions were removed, cells were washed with PBS, and 0.5 mg/mL MTT reagent (Thermo Fisher Scientific, Waltham, Massachusetts, United States) diluted in full culture medium was added. The cells were incubated for an hour, then the resulting formazan crystals were dissolved in dimethyl sulfoxide (DMSO, Molar Chemicals Kft, Halásztelek, Hungary), and the absorbance of the samples was assessed using a Synergy HTX plate reader (BioTek, Biotek Instruments lnc, Winooski, VT, United States) set at 570 nm. The absorbance values were normalized to the absorbance of the untreated control sample. The absorbance of the untreated control sample was considered as 100% viability.

### 2.17 LDH assay

The cytotoxic effect of NCR147_25-36_ W_33,35_/W^F^ peptide on MRC-5 fibroblast cells was measured by lactate-dehydrogenase (LDH) assay using Cayman LDH Cytotoxicity Assay Kit (Cayman Chemicals, Ann Arbor, Michigan, USA). For this, 10^4^ A549 and MRC-5 cells/well were seeded into 96-well plates and left to grow. After 24 h, cells were treated with 0, 6.25, 12.5, and 25 μM of NCR147_25-36_ W_33,35_/W^F^ diluted in serum-free medium. As a positive control 10% Triton-X-100 solution was applied, provided by the Cayman LDH Cytotoxicity Assay Kit, according to the manufacturer’s instructions. After 24 h of incubation, 100 μL of supernatant was transferred to another 96-well plate and incubated with the LDH Reaction Solution prepared according to the instructions of the manufacturer for 30 min at 37\u00B0C. The absorbance of the samples was measured with Synergy HTX plate reader (BioTek, Biotek Instruments lnc, Winooski, VT, United States) at 490 nm. The absorbance values of the samples were normalized to the absorbance of the untreated control.

### 2.18 Apoptosis detection

To detect whether apoptosis occurs upon the treatments, Apo-ONE Homogeneous Caspase-3/7 assay was performed. For this, 10^4^ MRC-5 cells/well were seeded into a 96-well plate. The next day, cells were treated with 0, 6.25, 12.5, and 25 μM of NCR147_25-36_ W_33,35_/W^F^ peptide for 24 h in serum-free DMEM. As a positive control, 5 μM of cisplatin treatment was performed for 24 h. After the incubation, Apo-ONE Caspase-3/7 Reagent, prepared according to the manufacturer’s instructions, was added to the samples in a 1:1 ratio. After 3 h of incubation, the fluorescence of the samples was measured at an excitation wavelength of 485/20 nm and an emission wavelength of 528/20 nm by Synergy HTX Plate reader (BioTek, Biotek Instruments lnc, Winooski, VT, United States). The fluorescence intensities were normalized to the fluorescence of the untreated control sample.

### 2.19 Statistical analysis

Statistical evaluation of the results was performed using GraphPad Prism 6 software, using One-way ANOVA, Dunnett’s multiple comparisons test.

### 2.20 Bioinformatic characterization of the peptides

3D structural predictions of the NCR147 peptide derivatives were generated using CSF ChimeraX version 1.9 (2024-12-11) in combination with ColabFold.

The physicochemical properties of peptides were predicted with tools available at the DBAASP property-calculation platform^2^ and the Peptalyzer server.^3^

### 2.21 Microscopy

#### 2.21.1 Confocal microscopy

To evaluate the effect of NCR147_25-36_ W_33,35_/W^F^ on *Cr. neoformans,* cells were grown for 16 h at 30 °C and then collected by centrifugation and washed twice with sterile distilled water. The cell concentrations were adjusted to 5×10^4^ cells/mL in YNB medium and were treated with NCR147_25-36_ W_33,35_/W^F^ at 1.56 (half-MIC) for 60 min. After treatment, cells were collected by centrifugation, rinsed with distilled water, and resuspended in 25 μL of distilled water.

Yeast cells were stained with 5 μM SYTO9 and 5 μM propidium iodide (PI). The fluorescence detection was performed using a Leica Stellaris 5 confocal laser scanning microscope (Leica Microsystems GmbH) with 63x oil immersion objective (N. A. 1.40).

#### 2.21.2 Scanning electron microscopy

5 μL of bacterial and yeast suspensions were spotted on 0.1 μm polycarbonate membrane filter (Merck) coated with 0.01% Poly-L-Lysine. The samples were fixed with 2.5% (v/v) glutaraldehyde in phosphate buffered saline (PBS, pH 7.4) for 1 h at 4 °C. The samples were washed twice with PBS and gradually dehydrated with ethanol series (10, 30, 50, 70, 80, 90, 96% and three times with 100% ethanol, each for minimum 30 min) at 4 °C. The samples were dried with a critical point dryer (K850: Quorum Technologies Ltd.), followed by 15 nm gold coating and observed under a JEOL JSM-7100F/LV scanning electron microscope.

## 3 Results

### 3.1 Design of NCR147 derivatives for the identification of sequences responsible for antimicrobial activity

The NCR147 sequence (AYIECEVDDDCPKPMKNSHPDTYYKCVKHRCQWAWK) comprises three distinct regions: a 12 amino acid long anionic N-terminal segment (AYIECEVDDDCP, pI 3.33), a 12 amino acids long mildly cationic central region (KPMKNSHPDTYY, pI 8.44), and a 12 amino acid long strongly cationic C-terminal segment (KCVKHRCQWAWK, pI 9.85) (Table 1). Since the antimicrobial activity of NCRs requires a cationic pI and a positive net charge, different versions of the 24 amino acid cationic C-terminal sequence with shorter lengths and amino acid substitutions were chemically synthesized with C-terminal amidation. The amino acid sequences and physicochemical properties of these peptides are presented in Table 1. NCR147_13-36_, NCR147_20-36_, NCR147_25-36_ and NCR147_28-36_, contain 24, 17, 12 and 9 amino acids long sequences from the C-terminal region, respectively. To investigate the role of cysteines and the need for disulfide bridge formation, we replaced the cysteines with serines in NCR147_25-36_C_26,31_/S and produced the oxidized form (NCR147_25-36_ox). The importance of tryptophan (W), which is putatively required for association with bacterial membranes, was tested by omitting the terminal WAWK sequence of NCR147_25-32_. In addition, the two W residues in NCR147_25-36_ were replaced individually and in both positions by alanine (A) (NCR147_25-36_ W_33_/A, NCR147_25-36_ W_35_/A, NCR147_25-36_ W_33,35_/A). Moreover, as fluorinated amino acids can enhance peptide stability and alter biological activities (Gottler et al., 2008; Marsh et al., 2009; Buer and Marsh, 2012; Arias et al., 2016; Feng et al., 2020) tryptophan was replaced with 5-fluoro-L-tryptophan in NCR147_25-36_ W_33_/W^F^, NCR147_25-36_ W_35_/ W^F^ and NCR147_25-36_ W_33,35_/ W^F^.

**Table 1:** Predicted physicochemical properties of NCR147 and its peptide derivatives.

ID	Sequence	μH_n_	H_n_	pI	DCP	AI	Z_7.4_	G	BI
NCR147	AYIECEVDDDCPKPMKNSHPDTYYKCVKHRCQWAWK	0.32	−0.05	7.61	−0.12	1.57	1.00	−1.17	2.59
NCR147_13-36_	KPMKNSHPDTYYKCVKHRCQWAWK	0.49	0.38	10.28	−0.45	2.04	5.80	−1.55	2.73
NCR147_20-36_	PDTYYKCVKHRCQWAWK	1.31	0.08	9.78	−0.34	2.36	3.76	−1.30	2.42
NCR147_25-36_	KCVKHRCQWAWK	1.28	0.69	10.82	−0.45	2.5	4.77	−1.14	2.39
NCR147_25-36_ox	KCVKHRCQWAWK	1.29	0.70	10.82	−0.46	2.5	4.77	−1.12	2.38
NCR147_25-36_C_26,31_/S	KSVKHRSQWAWK	1.21	0.91	14.00	−0.62	2.5	4.98	−1.69	3.17
NCR147_25-32_	KCVKHRCQ	1.35	1.24	10.54	−0.51	1.56	3.77	−1.23	3.70
NCR147_25-36_ W_33_/A	KCVKHRCQAAWK	1.29	0.72	10.82	−0.38	1.92	4.77	−0.92	2.44
NCR147_25-36_ W_35_/A	KCVKHRCQWAAK	1.28	0.72	10.82	−0.38	1.92	4.77	−0.92	2.44
NCR147_25-36_ W_33,35_/A	KCVKHRCQAAAK	1.29	0.75	10.82	−0.31	1.35	4.77	−1.23	2.48
NCR147_25-36_ W_33_/W^F^	KCVKHRCQW^F^AWK	1.30	0.71	10.82	−0.46	2.5	4.77	−1.12	2.41
NCR147_25-36_ W_35_/W^F^	KCVKHRCQWAW^F^K	1.30	0.71	10.82	−0.46	2.5	4.77	−1.12	2.41
NCR147_25-36_ W_33,35_/W^F^	KCVKHRCQW^F^AW^F^K	1.31	0.71	10.82	−0.46	2.5	4.77	−1.11	2.43
NCR147_28-36_	KHRCQWAWK	1.03	0.87	10.68	−0.66	2.93	3.87	−1.83	3.16

The table includes amino-acid sequences, normalized hydrophobic moment (μH_n_), normalized hydrophobicity (H_n_), isoelectric point (pI), disorder propensity (DCP), amphiphilicity index (AI), net charge at pH 7.4 (Z_7.4_), GRAVY score (G), and Boman index (BI in kcal/mol). Amino acids highlighted in red indicate amino acid substitutions in the peptide. The N-terminal anionic region of the NCR147 peptide (pI 3.33) is highlighted in magenta, while the strongly cationic C-terminal region (pI 9.85) is in turquoise. The physicochemical properties were predicted using tools available at the DBAASP property-calculation platform (https://dbaasp.org/toolspage=property-calculation) and the Peptalyzer server (https://peptidechemistry.org/peptalyzer/).

3D structural predictions of the NCR147 peptide derivatives generated with CSF ChimeraX version 1.9 in combination with ColabFold are presented in Supplementary Figure S3. Across the NCR147-derived series, the N-terminal truncations progressively remove acidic, polar residues and concentrate the peptide around the highly cationic, aromatic C-terminal motif. The full-length, only mildly net-positive AYIECEVDDDCPKPMKNSHPDTYYKCVKHRCQWAWK (NCR147) with four Cys can in principle form two disulfide bonds and a more compact, partly ordered fold. KPMKNSHPDTYYKCVKHRCQWAWK (NCR147_13-36_) and PDTYYKCVKHRCQWAWK (NCR147_20-36_) are shorter, more strongly cationic and amphipathic, still retaining two C residues that could form a single disulfide-stabilized loop, but overall are expected to be more flexible with only local structure around the C/W/Y cluster. KCVKHRCQWAWK (NCR147_25-36_) is a small, highly cationic fragment enriched in K/R/H and aromatics; consistent with the ColabFold prediction, it is intrinsically disordered in solution, sampling dynamic conformations with only transient turns around the conserved C–W region. 5-fluoro-L-tryptophane (W^F^) is a near-isosteric W analog. Swapping W to W^F^ will barely change the physicochemical properties. It may affect the local chemistry but not create a new fold or change the backbone and disordered conformation. Replacing the C-terminal WAW in KCVKHRCQWAWK with AAA to give KCVKHRCQAAAK (NCR147_25-36_ W_33,35_/A) keeps length and net positive charge essentially the same but removes both aromatic side chains and most of the hydrophobic character of that C-terminal segment, thus it is more polar, less amphipathic and lacks aromatics with weaker affinity for hydrophobic or membrane environments and disordered. KHRCQWAWK (NCR147_28-36_) is a further shortened, highly cationic, aromatic-rich peptide (K/H/R + two W). It’s strongly amphipathic: clustered basic residues plus a WAW hydrophobic patch, a flexible but “sticky” peptide that can interact well with membranes or protein surfaces. KCVKHRCQ (NCR147_25-32_) is similarly cationic but lacks aromatics and instead has two cysteines, it is a bit less hydrophobic and less membrane-interactive and disordered as a free peptide, but it’s better suited as a cysteine-rich “anchor” or crosslinking motif.

The antibacterial effectiveness of these peptides was tested on thirteen Gram-negative and three Gram-positive bacteria comprising both human pathogens: *Escherichia coli* (*Ec*), *Salmonella enterica* (*Se*), *Klebsiella pneumoniae* (*Kp*), *Acinetobacter baumannii* (*Ab*), *Pseudomonas aeruginosa* (*Pa*), *Enterococcus faecalis* (*Ef*), *Listeria monocytogenes* (*Lm*), *Staphylococcus aureus* (*Sa*), and plant pathogens; *Pseudomonas syringae* pv. *tomato* (*Pstm*), *Pseudomonas syringae* pv. *tabaci* (*Pstb*), *Pseudomonas syringae* pv. *maculicola* (*Psm*), *Burkholderia gladioli* (*Bg*), *Xanthomonas citri* pv. *malvacearum* (*Xc*), *Dickeya chrysanthemi* (*Dc*), *Pectobacterium atrosepticum* (Pat), *Agrobacterium tumefaciens* (At) (Table 2). The Minimum Bactericidal Concentrations (MBC) were determined after 3 h of treatment of 10^5^ log phase bacteria with two-fold serial dilutions of the peptides from 100 μM to 1.6 μM concentrations in 20 mM Potassium Phosphate Buffer pH 7.4 (PPB) and followed by monitoring the growth of bacteria on LB agar plates. Under these experimental conditions, full-length, NCR147 showed only weak antibacterial activity against *Lm* and *Xc*, and slightly stronger against *Pstb* and *Psm*. The absence of the anionic N-terminal 12 amino acids in NCR147_13-36_ increased the antibacterial activity against several bacteria with MBC values of 6.25–12.5 μM and was remarkable active against *Psm* with 1.6 μM MBC. The lack of 19 N-terminal amino acids in NCR147_20-36_ provoked variable results, generally less efficiency with higher MBC values on human pathogens, except for *Ef, Ab, Lm* while among plant pathogens *Pstb*, *Xc* and *Dc* became significantly more sensitive.

**Table 2:** Antimicrobial activities of the NCR147 peptide derivatives against human and plant pathogens.

Peptides	MBC of peptides in *μM* against Gram-negative and Gram-positive pathogens															
	*Ec*	*Se*	*Kp*	*Ab*	*Pa*	*Ef*	*Lm*	*Sa*	*Pstm*	*Pstb*	*Psm*	*Bg*	*Xc*	*Dc*	*Pat*	*At*
NCR147	50	50	>50	50	50	>50	25	>50	>50	12.5	**6.25**	>50	25	>50	>50	>50
NCR147_13-36_	12.5	50	>100	12.5	**6.25**	50	12.5	50	12.5	**6.25**	**1.6**	>50	12.5	>50	12.5	>50
NCR147_20-36_	50	>100	>100	12.5	25	12.5	**6.25**	>100	25	**6.25**	12.5	>50	**6.25**	**3.13**	50	>50
NCR147_25-36_	25	50	>50	25	25	>50	>50	50	12.5	**3.13**	**3.13**	>50	25	>50	12.5	>50
NCR147_25-36_ox	25	50	>100	25	25	>100	25	50	**6.25**	**3.13**	**6.25**	>50	**6.25**	**6.25**	12.5	>50
NCR147_25-36_C_26,31_/S	25	>100	>100	>100	25	>100	>100	>100	100	**6.25**	25	>50	>100	>50	>50	>50
NCR147_25-32_	>50	>50	>50	>50	>50	>50	>50	>50	>50	>50	**6.25**	>50	>50	>50	>50	>50
NCR147_25-36_ W_33_/A	>50	>50	>50	>50	>50	>50	>50	>50	**6.25**	**6.25**	**3.13**	>50	>50	>50	>50	>50
NCR147_25-36_ W_35_/A	>50	>50	>50	>50	>50	>50	>50	>50	**6.25**	**6.25**	**6.25**	>50	>50	25	>50	>50
NCR147_25-36_ W_33,35_/A	>50	>50	>50	>50	>50	>50	>50	>50	**6.25**	**6.25**	**3.13**	>50	>50	25	>50	>50
NCR147_25-36_ W_33_/W^F^	12.5	12.5	>50	25	12.5	>50	50	12.5	12.5	**3.13**	**6.25**	>50	12.5	>50	12.5	>50
NCR147_25-36_ W_35_/W^F^	**6.25**	**6.25**	>50	**6.25**	**6.25**	>50	12.5	**6.25**	**3.13**	**1.6**	**1.6**	>50	**6.25**	>50	**6.25**	>50
NCR147_25-36_ W_33,35_/W^F^	**3.13**	**3.13**	>50	**3.13**	**6.25**	>50	**3.13**	**3.13**	**3.13**	**1.6**	**3.13**	>50	**3.13**	>50	12.5	>50
NCR147_28-36_	**6.25**	25	>100	**6.25**	12.5	>100	>100	25	**6.25**	**6.25**	**3.13**	>50	**6.25**	>50	25	>50

Minimum bactericidal concentration (MBC, in μM) of peptides against *Escherichia coli* (*Ec*), *Salmonella enterica* (*Se*), *Klebsiella pneumoniae* (*Kp*), *Acinetobacter baumannii* (*Ab*), *Pseudomonas aeruginosa* (*Pa*), *Enterococcus faecalis* (*Ef*), *Listeria monocytogenes* (*Lm*), *Staphylococcus aureus* (*Sa*), *Pseudomonas syringae* pv. *tomato* DC3000 (*Pstm*), *Pseudomonas syringae* pv. *tabaci* (*Pstb*), *Pseudomonas syringae* pv. *maculicola* (*Psm*)*, Burkholderia gladioli* (*Bg*), *Xanthomonas citri* pv. *malvacearum* (*Xc*), *Dickeya chrysanthemi* (*Dc*), *Pectobacterium atrosepticum* (Pat), *Agrobacterium tumefaciens* (*At*) after 3 h of treatment in 20 mM PPB. The gray background behind the strain abbreviation indicates Gram-negative bacteria. MBCs from 25 μM to 1.6 μM are shown with increasing darkness of gray backgrounds. Bold values indicate stronger activities (0.8, 1.6, 3.13, 6.25 μM).

In the absence of the first 24 amino acids, the antimicrobial activity was preserved in NCR147_25-36_ against *Pa* and *Ab*, while the MBC values were lower on some tested *Ps* strains, and this shorter peptide was more effective against *Pat* than NCR147_20-36_. Neither NCR147_25-36_ nor its derivatives were effective against *Kp*, *Ef*, *Bg*, *Dc* and *At* while the longer peptide, NCR147_20-36_ was able to kill *Ef* and *Dc* indicating the need for the extra 5 amino acids for bactericidal effect in the case of these species. The activity of the reduced and oxidized NCR147_25-36_ peptides was similar on the human and on most plant pathogens though the oxidized form was somewhat more active against *Lm*, *Pstm*, *Xc* and *Dc* compared to the reduced NCR147_25-36_. Substitution of the cysteine residues with serine had a larger negative effect in the case of *Ab*, *Lm*, *Pstm, Xc* and *Dc* while other bacteria were less (*Se*, *Sa*, *Pstb*, *Psm* and *Pat*) or not affected. Alanine substitution of tryptophan abolished the antimicrobial activity against all tested bacteria, except for the *Ps* strains. Replacement of W with 5-fluoro-L-tryptophan (W^F^) in NCR147_25-36_ improved the antibacterial efficacy against many of the tested strains. W^F^ substitution of W_33_ was less effective than that of W_35_ and replacement of both W_33_ and W_35_ with W^F^ increased most the antibacterial activity of the sensitive strains and resulted in as low as 1.6 *μM* MBC. NCR147_28-36_ retained the antibacterial activity against most bacteria, while the 8 amino acids long NCR147_25-32_ lacking the terminal WAWK sequence was inactive against all strains except for *Psm,* the most sensitive strain among the tested ones. NCR147_28-36_, the 9 amino acids long sequence from the C-terminus also retained antibacterial activity against several pathogens tested, except *Kp*, *Ef*, *Lm*, *Bg*, *Dc* and *At.* These studies, in line with the physicochemical characteristics of peptides, confirmed that the antibacterial activity is embedded in the C-terminal amphipathic region comprising clustered basic residues and the WAW hydrophobic patch. Replacement of tryptophan with 5-fluoro-L-tryptophan substantially broadened the antimicrobial spectrum and increased potency, with activity extending to 11 of the 15 species tested.

Scanning electron microscopy of *A. baumannii* treated with NCR147_25-36_ and NCR147_25-36_ W_33,35_/W^F^ revealed marked alteration of the bacterial surface, including pronounced blebbing and cellular swelling (Supplementary Figure S4). Treatment with NCR147_25-36_ W_33,35_/W^F^ additionally resulted in frequent cell collapse, indicating more severe damage of cells.

### 3.2 Effects of the NCR147 peptide derivatives on the formation and eradication of biofilms

Biofilm formation at bacterial infections is a key mechanism by which bacteria persist in the body, making infections harder to treat. Here we investigated how the NCR147 derivatives affect biofilm formation of *Acinetobacter baumannii*, the number one bacterial species on the WHO pathogen list (WHO Bacterial Priority Pathogens List, 2024; WHO, 2024). As the biofilms were formed in 1/10 Mueller Hinton Broth (MHB) and the composition of media can influence the antimicrobial activity of peptides, first, we determined the MBCs of the NCR147 derivatives and three antibiotics, commonly used against *Acinetobacter* infections in 1/10 MHB (Table 3). The antibiotics were cefepime, a fourth-generation cephalosporin, levofloxacin, a broad-spectrum third-generation fluoroquinolone, and meropenem, a beta-lactam antibiotic. The MBCs of peptides in PPB and MHB were either the same or differed only with one dilution step (twice lower or higher concentrations). The W^F^ substitution in W_35_ position or at both W_33_ and W_35_ positions was particularly effective. Among the antibiotics, cefepime was inactive, while levofloxacin was very effective (Table 3).

**Figure 1:**
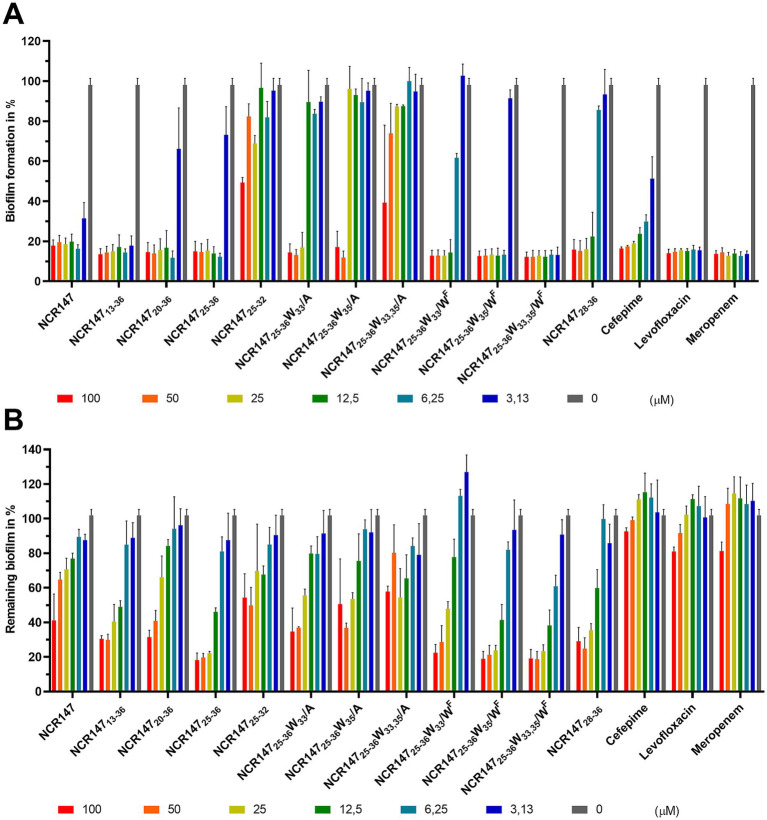
Antibiofilm effects of NCR147 peptide derivatives and antibiotics against *A. baumannii*. **(A)** Inhibition of biofilm formation by the peptides and antibiotics. **(B)** Biofilm disruption by the peptides and antibiotics. The untreated control is set as the baseline at 100%. The bars represent the relative quantity of biofilm formed in **(A)** and the residual biofilm in **(B)** compared to the control. The experiment was performed with three biological replicates, and the figures illustrate the mean ± SD.

To evaluate the effects of NCR147 derivatives and the above antibiotics on biofilm formation (Figure 1A) *A. baumannii* was cultivated in 1/10 MHB for 24 h in the absence or presence of peptides or antibiotics in a concentration range from 100 μM to 0.8 μM. All three antibiotics (cefepime, levofloxacin and meropenem) inhibited biofilm formation at 3.13 μM and higher concentrations. Among the NCR147 derivatives those containing one or two W^F^ residues were the most active ones at 3.13 μM (NCR147_25-36_ W_35_/W^F^, NCR147_25-36_ W_33,35_/W^F^). NCR147, NCR147_13-36_, NCR147_20-36_, NCR147_25-36_ and NCR147_25-36_ W_33_/W^F^ inhibited biofilm formation at 6.25–12.5 μM. Replacement of tryptophan with alanine (W/A) removed the inhibitory effect on biofilm formation below 25 μM concentrations.

**Table 3:** Minimum bactericidal concentrations (MBC, in μM) of the NCR147 peptide derivatives and antibiotics against *A. baumannii* in PPB and 1/10 MHB.

NCRs/antibiotics	MBC (in PPB)	MBC (in MHB)
NCR147	50	100
NCR147_13-36_	12.5	**6.25**
NCR147_20-36_	12.5	12.5
NCR147_25-36_	25	12.5
NCR147_25-36_ox	25	>100
NCR147_25-36_C_26,31_/S	>100	>100
NCR147_25-32_	>50	>100
NCR147_25-36_ W_33_/A	>50	50
NCR147_25-36_ W_35_/A	>50	50
NCR147_25-36_ W_33,35_/A	>50	100
NCR147_25-36_ W_33_/W^F^	25	12.5
NCR147_25-36_ W_35_/W^F^	**6.25**	**6.25**
NCR147_25-36_ W_33,35_/W^F^	**3.13**	**6.25**
NCR147_28-36_	**6.25**	12.5
Cefepime	>100	>100
Levofloxacin	**0.8**	6.25
Meropenem	25	25

Increasing background color intensities mark higher effectiveness of antibacterial activity. Bold values indicate stronger activities (0.8, 1.6, 3.13, 6.25 μM).

Biofilms are typically highly resistant to antimicrobial agents. To evaluate whether the antibiotics and NCR147 peptide derivatives could effectively eradicate biofilms, *A. baumannii* was cultivated in 1/10 MHB for 24 h and subsequently treated with these agents at concentrations of up to 100 μM for 3 h. None of the antibiotics tested, and none of the peptides below 25 μM, were successful in eradicating the existing biofilms, except NCR147_25-36_ W_35_/W^F^ and NCR147_25-36_ W_33,35_/W^F^ achieving approximately 50% biofilm degradation at a concentration of 12.5 μM (Figure 1B). At concentrations of 50 μM and 100 μM, all peptides except NCR147_25-32_ were effective in reducing the biofilms, leaving only 30–50% of the biofilm intact, whereas the antibiotics remained ineffective.

### 3.3 Killing kinetics of NCR147 peptide derivatives

**Figure 2:**
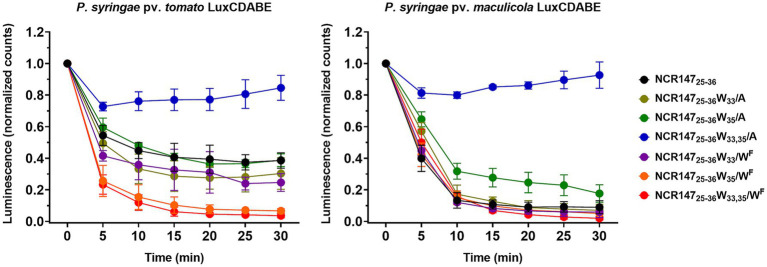
Expression of the *luxCDABE* operon in *P. syringae* pv. *tomato* DC3000 *and P. syringae* pv. *maculicola* strains treated with NCR147_25-36_ and its A/W^F^ and W/W^F^ substituted derivatives. The experiment was performed with three biological replicates, and the figures illustrate the mean ± SD.

Antimicrobial cationic NCR peptides are known for their rapid microbicidal effects, often killing microbes within minutes (Jenei et al., 2020). To explore the cessation of vital bacterial functions, *Pseudomonas syringae* pv. *tomato* and *Pseudomonas syringae* pv. *maculicola*, both expressing the *luxCDABE* operon constitutively, were used to monitor transcriptional activity via luminescence measurements. Luminescence was measured in PPB every 5 min over a 30-min period in the presence of 20 μM NCR147_25-36_, and its alanine or 5-fluoro-L-tryptophan substituted variants (NCR147_25-36_ W_33_/A, NCR147_25-36_ W_35_/A, NCR147_25-36_ W_33,35_/A and NCR147_25-36_ W_33_/W^F^, NCR147_25-36_ W_35_/W^F^, NCR147_25-36_ W_33,35_/W^F^) and then bacterial survival was assessed by colony formation on agar plates. All peptides, except NCR147_25-36_ W_33,35_/A, caused a rapid decrease in luminescence in both bacterial strains within 5 min (Figure 2) and inhibited growth on agar plates, confirming their fast bactericidal effect. NCR147_25-36_ W_33,35_/A had a moderate effect on *luxCDABE* expression, with luminescence remaining high during treatment, while the treated bacteria were unable to grow on agar plates, suggesting a viable but non-culturable (VBNC) state.

### 3.4 Interaction of NCR147_25-36_ and its 5-fluoro-L-tryptophan variant (NCR147_25-36_ W_33,35_/W^F^) with bacterial membranes

#### 3.4.1 Interaction with membrane lipids

**Figure 3:**
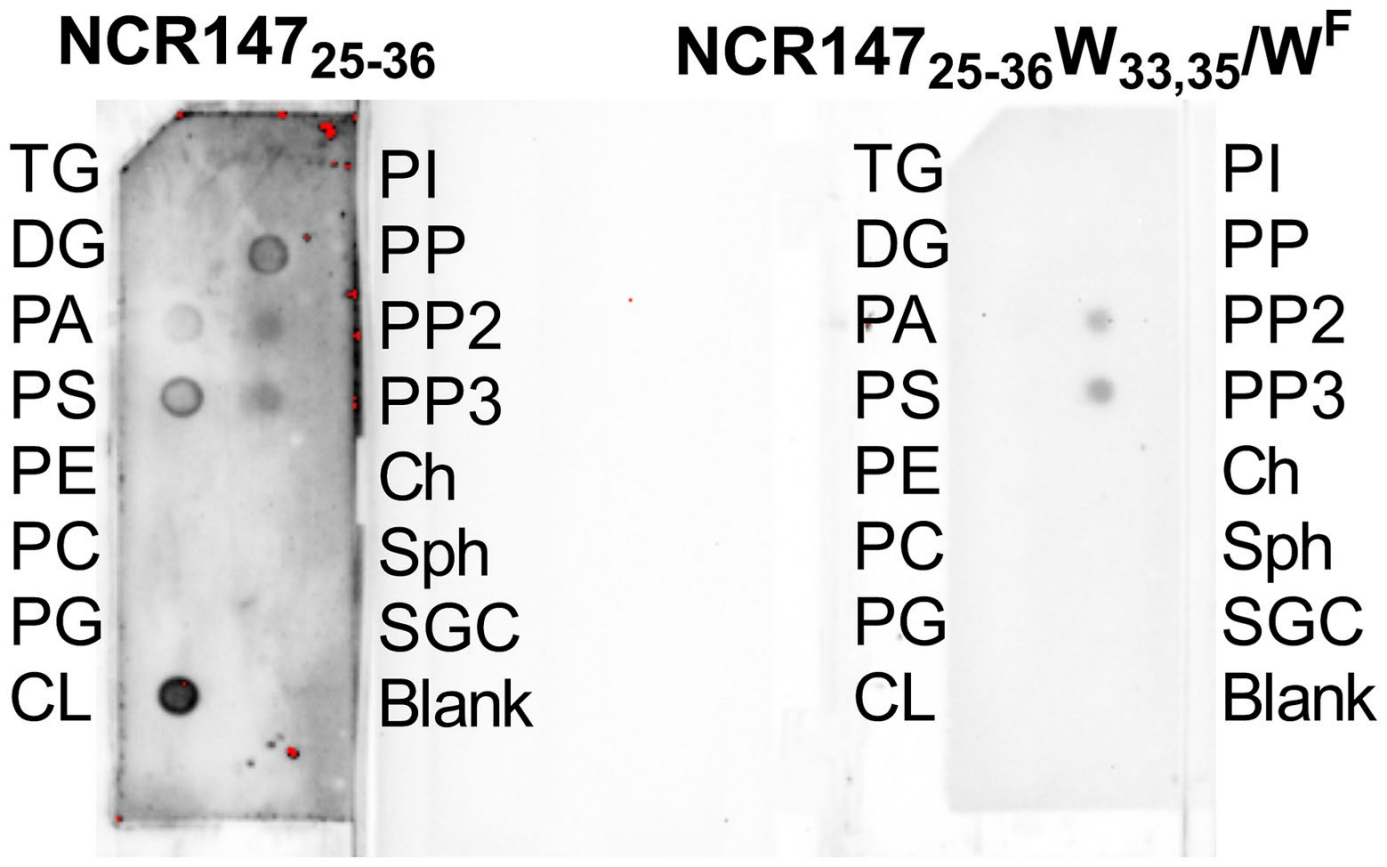
Binding of NCR147_25-36_ and NCR147_25-36_ W_33,35_/W^F^ to membrane lipids. TG: Triglyceride, DG: Diacylglycerol, PA: Phosphatidic acid, PS: Phosphatidylserine, PE: Phosphatidylethanolamine, PC: Phosphatidylcholine, PG: Phosphatidylglycerol, CL: Cardiolipin, PI: Phosphatidylinositol, PP: PtdIns-4-P, PP2: PtdIns-4,5-P2, PP3: PtdIns-,4,5-P3, Ch: Cholesterol, Sph: Sphingomyelin, SGC: 3-Sulfogalactosylceramide.

The amino acid sequences of NCR147_25-36_ and NCR147_25-36_ W_33,35_/W^F^ differ solely by the W/W^F^ substitution, which significantly broadened the spectrum of activity against pathogens. These findings indicate that even the initial interactions with the bacterial surface might vary between the two peptides. Lipid strips containing various membrane lipid components were treated with StrepII-tagged peptides, and the presence of bound peptides was detected using a StrepTactin–horseradish peroxidase (HRP) conjugate. NCR147_25-36_ demonstrated strong binding to cardiolipin, moderate binding to phosphatidylinositol-4-phosphate (PtdIns-4-P) and phosphatidylserine, and weak binding to phosphatidic acid, phosphatidylinositol-4,5-bisphosphate (PtdIns-4,5-P2), and phosphatidylinositol-3,4,5-trisphosphate (PtdIns-3,4,5-P3). In contrast, W/W^F^ substitution in NCR147_25-36_ W_33,35_/W^F^ abolished the cardiolipin binding and exhibited only weak interactions with PtdIns-4,5-P2 and PtdIns-3,4,5-P3, suggesting that the W/W^F^ substitution alters the peptide’s mode of action (Figure 3).

#### 3.4.2 Effects on bacterial efflux pumps

**Figure 4:**
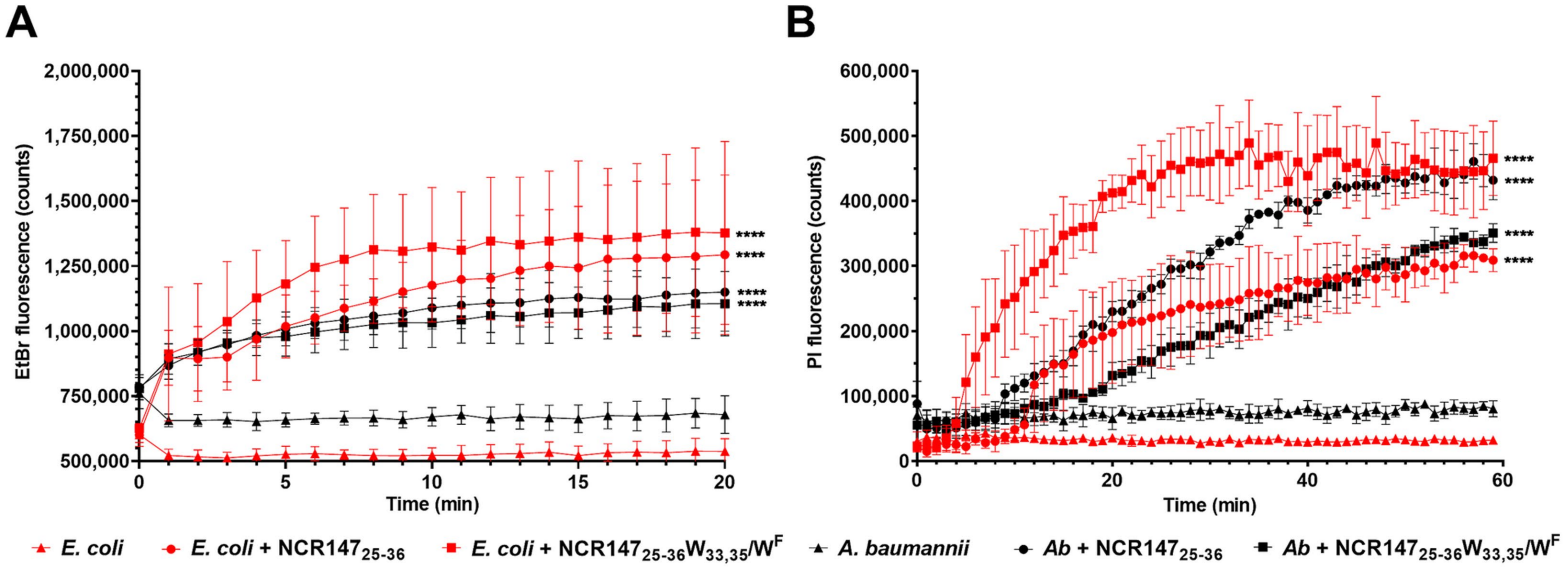
Effects of 6.25 μM NCR147_25-36_ and NCR147_25-36_ W_33,35_/W^F^ on **(A)** the efflux pumps and **(B)** membrane permeability in OD_600_ = 0.1 *E. coli* and *A. baumannii*. **(A)** Fluorescence of intracellular EtBr. **(B)** Intracellular PI fluorescence. One-way ANOVA was performed to compare NCR treated samples to untreated samples across the time course, *p* < 0.0001 (****). The experiment was performed with three biological replicates, and the figures illustrate the mean ± SD.

The peptides’ binding to bacterial membrane lipids may also influence the activity of efflux pumps, which protect bacteria by expelling harmful molecules into the periplasm, where they are transported out of the cell via outer membrane porins. The efflux pump activity was monitored in *E. coli* and *A. baumannii* preloaded with ethidium bromide (EtBr) and then left untreated or treated with 6.25 μM NCR147_25-36_ or NCR147_25-36_ W_33,35_/W^F^. The peptides were applied to bacterial cultures at 10 times higher cell density than for the determination of their minimum bactericidal concentration (MBC). This setup ensured that the treatment was administered at a sublethal dose. Efflux pump activity was assessed by measuring EtBr fluorescence over 20 min (Figure 4A). In untreated control bacteria, fluorescence remained constant, whereas the presence of peptides caused an increase in fluorescence, indicating efflux pump inhibition. The inhibition was more pronounced in *E. coli* than in *A. baumannii*, and NCR147_25-36_ W_33,35_/W^F^ exhibited slightly greater inhibitory activity compared to NCR147_25-36_.

#### 3.4.3 Effect on membrane permeability

Bacterial membranes are the most probable primary targets of AMPs and cationic NCRs. Membrane permeabilization and damage were assessed using propidium iodide (PI), a membrane-impermeable cationic dye that does not enter living cells but penetrates damaged membranes and accumulates within bacterial cells. Once inside, PI binds to DNA and RNA, resulting in bright fluorescence. Treatment of OD_600_ = 0.1 *E. coli* and *A. baumannii* with NCR147_25-36_ and NCR147_25-36_ W_33,35_/W^F^ at 6.25 μM resulted in fast accumulation of PI (Figure 4B). NCR147_25-36_ W_33,35_/W^F^ provoked more pronounced and faster membrane damage than NCR147_25-36_, particularly in *E. coli*.

### 3.5 Bacterial protein targets of NCR147_25-36_ and NCR147_25-36_ W_33,35_/W^F^

To identify potential protein partners of NCR147_25-36_ and NCR147_25-36_ W_33,35_/W^F^, the peptides were synthetized with C-terminal StrepII tag (WSHPQFEK). NCR147_25-36_-StrepII, NCR147_25-36_ W_33,35_/W^F^-StrepII and StrepII alone (as a control) were incubated with crude protein extracts of *E. coli*. Peptide-protein complexes were isolated using affinity chromatography on Strep-Tactin resin beads, and bound proteins were separated and identified via liquid chromatography-mass spectrometry (LC–MS/MS). The analysis revealed 7 proteins binding to the StrepII control, 204 proteins binding to NCR147_25-36_-StrepII, and 214 proteins binding to NCR147_25-36_ W_33,35_/W^F^-StrepII (Supplementary Table S1: Protein interaction partners of NCR147 derivatives in *E. coli*). Among these, 36 proteins were unique to NCR147_25-36_-StrepII, while 47 were unique to NCR147_25-36_ W_33,35_/W^F^-StrepII.

Key common interactors included DNA-directed RNA polymerase subunits *β* and β’ (RpoB and RpoC), the 30S ribosomal subunit S1 (RpsA), and dihydrolipoyllysine residue acetyltransferase, the E2 component of the pyruvate dehydrogenase complex (AceF). Additional shared proteins with high peptide counts included dihydrolipoyllysine residue succinyltransferase (SucB) and dihydrolipoyl dehydrogenase (LpdA), components of the 2-oxoglutarate dehydrogenase complex and pyruvate dehydrogenase complex, respectively. Numerous ribosomal proteins were also identified, such as L1, L2, L15, L22, S3, S4, S5, S7 and S9, with higher representation in the NCR147_25-36_ W_33,35_/W^F^-StrepII complexes compared to NCR147_25-36_-StrepII. Other notable proteins identified included translational initiation factor IF2 (InfB), elongation factor Tu (TufA), DNA-directed RNA polymerase subunit *α* (RpoA), polyribonucleotide nucleotidyltransferase (Pnp), outer membrane proteins (OmpA, OmpC, OmpF), the E1 component of the pyruvate dehydrogenase complex (AceE), acetyl-CoA carboxylase (AccA; the first enzyme in lipid synthesis), ADP-L-glycero-D-mannoheptose-6-epimerase (HldD; involved in lipopolysaccharide core phosphorylation), and several chaperones such as DnaK, GroEL and ProQ. Cell division proteins (DamX, FtsZ, MreB) were also identified but only with low peptide counts. It is worth to note that some proteins, including DnaK, elongation factor Tu, and dihydrolipoyl dehydrogenase, were also detected in the StrepII control, albeit at much lower levels.

### 3.6 Antifungal activity of NCR147_25-36_ W_33,35_/W^F^

Antimicrobial peptides (AMPs) often exhibit both antibacterial and antifungal activities. Given the broad-spectrum antibacterial properties of NCR147_25-36_ W_33,35_/W^F^, we evaluated its potential antifungal activity against *Candida albicans* and *Cryptococcus neoformans*. Minimum inhibitory concentrations (MICs) were determined using a broth microdilution assay in Yeast Nitrogen Base (YNB) medium, with peptide concentrations ranging from 1.56 μM to 25 μM. NCR147_25-36_ W_33,35_/W^F^ showed clear antifungal activity, effectively inhibiting the growth of both fungal pathogens as the MIC against *C. albicans* was 12.5 μM, while *Cr. neoformans* was more susceptible, with a MIC of 3.1 μM (Supplementary Figure S5).

Scanning electron microscopy (SEM) was performed to elucidate the morphological alterations caused by NCR147_25-36_ W_33,35_/W^F^-treatment on *Cr. neoformans*. Unlike the control cells, *Cr. neoformans* cells exposed to 1.56 μM NCR147_25-36_ W_33,35_/W^F^ for 60 min exhibited structural alterations, including cellular collapse (Supplementary Figures S6A,E). Confocal microscopy further supported these findings: control samples showed almost only SYTO9-stained green live cells whereas peptide-treated cells were stained by both SYTO9 and PI which enters dead cells and produce red fluorescence (Supplementary Figures S6B–D, vs. F–H). This dual staining indicates loss of membrane integrity and confirms that the peptide treatment resulted in loss of cell viability.

### 3.7 Cytotoxicity of NCR147_25-36_ W_33,35_/W^F^ on human cells

#### 3.7.1 Cytotoxicity of NCR147_25-36_ W_33,35_/W^F^ on human red blood cells

**Figure 5:**
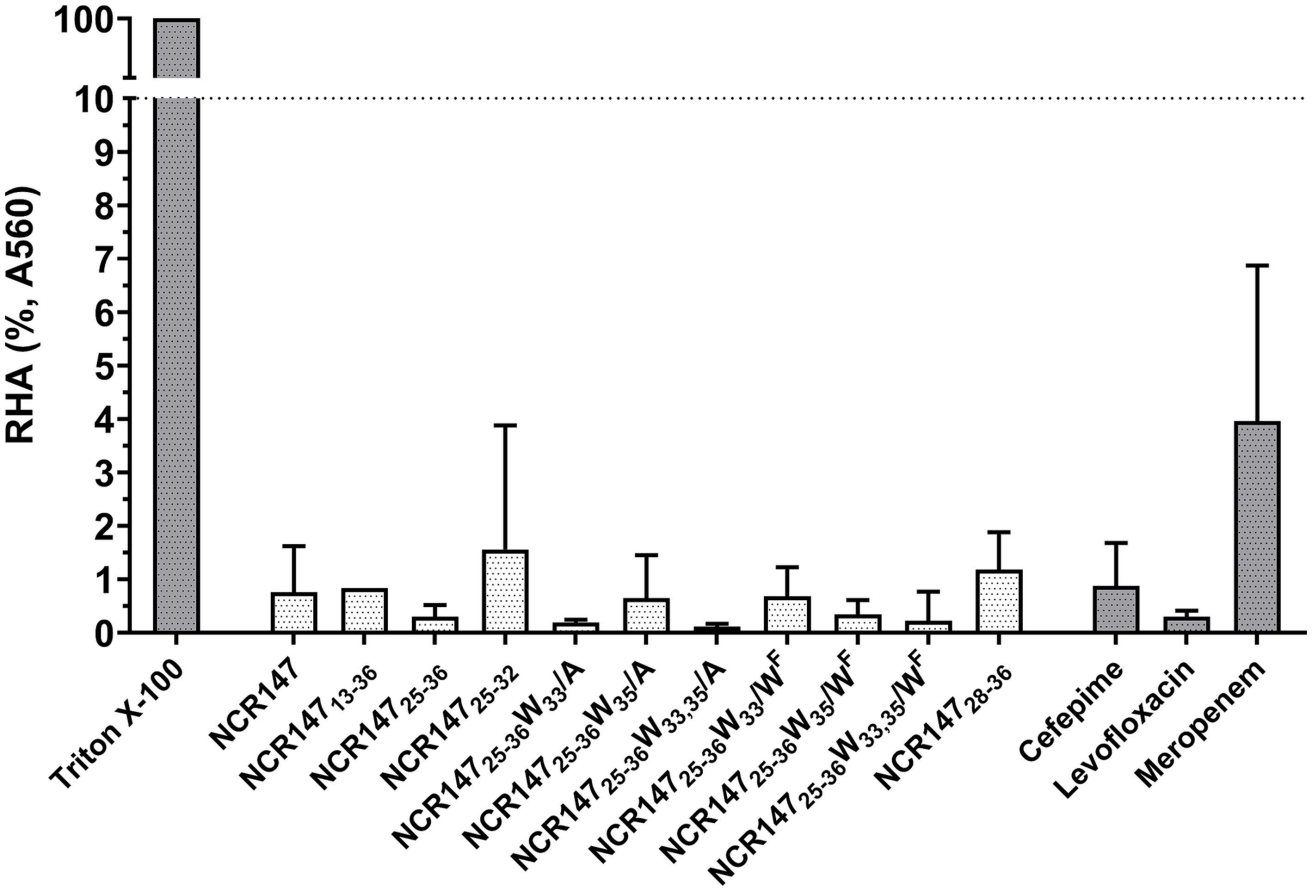
Hemolysis of human red blood cells in the presence of TritonX-100 and NCR147 derivatives and antibiotics (100 μM). The values represent the means from two independent experiments. The bars indicate standard deviations. For statistical analysis one-way ANOVA was used, *p*-value < 0.0001 (****), compared to Triton X-100. RHA = Relative hemolytic activity. The experiment was performed with three biological replicates, and the figures illustrate the mean ± SD.

The observed membrane interactions raised the concern that the peptides might also damage human red blood cells (HRBC) and induce hemolysis. To assess potential side effects of the active peptides, hemolysis assay was conducted. Human red blood cells were treated with the NCR147 derivatives and the three antibiotics, each at a concentration of 100 μM for 1 h. Triton X-100 was used as a positive control, achieving 100% hemolysis. None of the peptides or antibiotics caused notable hemolysis, even at this high concentration, indicating their safety for HRBC (Figure 5).

#### 3.7.2 Effect of NCR147_25-36_ W_33,35_/W^F^ on the viability of human cells

**Figure 6:**
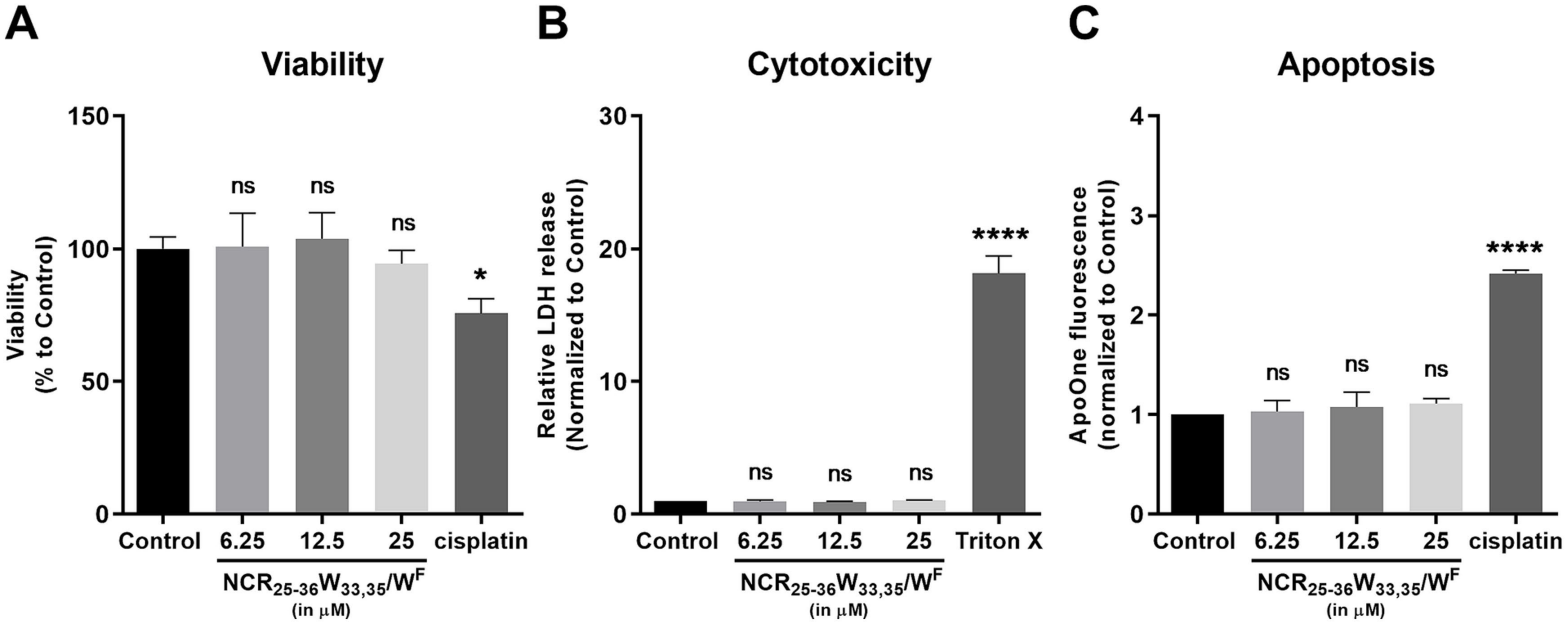
Cytotoxic and apoptotic effects of the NCR147_25-36_ W_33,35_/W^F^ peptide in MRC-5 cells. **(A)** Viability of MRC-5 cells in the presence of the applied concentration of NCR147_25-36_ W_33,35_/W^F^ peptide was not affected. As a positive control, 5 μM cisplatin treatment was applied. **(B)** Cytotoxicity of NCR147_25-36_ W_33,35_/W^F^ on MRC-5 cells. Treatment with NCR147_25-36_ W_33,35_/W^F^ did not induce cytotoxicity in MRC-5 cells relative to untreated controls. Triton X-100 was used as a positive control. **(C)** Apoptosis induction by the NCR147_25-36_ W_33,35_/W^F^ peptide in MRC-5 cells. No apoptosis was observed in MRC-5 cells upon peptide treatment. 5 μM cisplatin served as a positive control. All data were analyzed using one-way ANOVA followed by Dunnett’s multiple comparisons test; ****p < 0.0001.

Since NCR147_25-36_ W_33,35_/W^F^ exhibited toxicity toward bacterial and fungal species, we investigated whether the peptide also affects the viability of human cells. Non-cancerous MRC-5 human fibroblast cells were treated with NCR147_25-36_ W_33,35_/W^F^ at concentrations of 6.25, 12.5 and 25 μM, alongside cisplatin at 5 μM as a positive control. Cell viability was assessed using the MTT assay after 24 h of treatment (Figure 6A). The NCR147_25-36_ W_33,35_/W^F^ did not significantly affect the viability of MRC-5 fibroblast cells compared to untreated controls. Cisplatin slightly decreased the viability of MRC-5 cells.

#### 3.7.3 Cytotoxicity and apoptosis-inducing effect of NCR147_25-36_ W_33,35_/W^F^ in MRC-5 human fibroblast cells

To assess the cytotoxic effect of NCR147_25-36_ W_33,35_/W^F^, lactate dehydrogenase (LDH) release was measured in the culture supernatants. None of the applied concentrations of the peptide induced a significant increase in LDH levels compared to untreated control cells, indicating that the peptide did not cause membrane disruption, cell lysis, or necrotic cell death. In contrast, treatment with the positive control (Triton X-100) resulted in a significant elevation of LDH activity (Figure 6B).

To determine whether the NCR147_25-36_ W_33,35_/W^F^ peptide induces apoptosis, caspase-3/7 activity was measured in MRC-5 cells. No increase in caspase activity was detected in MRC-5 cells. As expected, the positive control (5 μM cisplatin) elicited the highest level of caspase-3/7 activation (Figure 6C).

## 4 Discussion

In this work we show that the antimicrobial activity of the neutral NCR147 peptide from *M. truncatula* relies on its cationic C-terminal region. When tested against 16 pathogenic bacterial species, only three—*Klebsiella pneumoniae*, *Burkholderia gladioli* and *Agrobacterium tumefaciens*—displayed resistance to the NCR147 derivatives. Notably, the effectiveness of the 24-, 17- and 12-amino-acid C-terminal fragments (NCR147_13-36_, NCR147_20-36_ and NCR147_25-36_) differed despite all of them carrying high pI values and positive net charges between +3,76 and +5,8 (Table 1). This observation is consistent with our previous findings showing that, beyond high pI and positive net charge, the specific amino acid composition of NCR peptides also plays a critical role in determining antimicrobial activity (Lima et al., 2022).

Unlike the structurally related plant defensins, which depend on four disulfide bridges for stability and activity, NCR peptides can remain bioactive even in their reduced, linear form. All C-terminal fragments of NCR147 from position 25 onward exhibit intrinsically disordered structures, suggesting that this conformational flexibility may support efficient interaction with microbial membranes. Their cationic nature further promotes electrostatic attraction to the negatively charged bacterial surface, providing a key initial step for antimicrobial activity.

Tryptophan residues appear to contribute an additional and essential layer to this mechanism. The bulky aromatic indole side chain of tryptophan has a strong propensity to partition into the membrane interfacial region, enabling interactions mediated by hydrophobic contacts, cation-*π* interactions of W or W^F^ with K, R and H likely influencing membrane anchoring and antimicrobial potency. Through these combined properties, tryptophan can anchor peptides to microbial membranes and facilitate membrane permeabilization, destabilization or translocation. These characteristics commonly make tryptophan-rich or tryptophan-containing antimicrobial peptides particularly potent. In line with this, our results highlight the importance of the C-terminal WAW hydrophobic patch in the antimicrobial activity of the NCR147 derivatives.

*Pseudomonas aeruginosa* was more sensitive to the 24 amino acids long sequence than to the shorter derivatives. *Enterococcus faecalis* was only sensitive to the 17 amino acids long sequence that was also more active against *Listeria monocytogenes*, *Xanthomonas citri* pv. *malvacearum* and *Dickeya chrysanthemi*. The 12 amino acids long C-terminal sequence (NCR147_25-36_) was mostly active against the plant pathogens. The reduced and oxidized forms of NCR147_25-36_ had similar activities, only *Xanthomonas citri* pv. *malvacearum* and *Dickeya chrysanthemi* exhibited higher sensitivity for the oxidized form. The substitution of cysteine with serine had either no effect or only slightly reduced the activity. The substitution of tryptophan with alanine (W/A) abolished the activity against all bacteria, except the *Pseudomonas syringae* strains, supporting the essential contribution of tryptophan in the WAWK sequence to the antimicrobial activity. Accordingly, deletion of the C-terminal WAWK sequence (NCR147_25-32_) abolished the antimicrobial activity against all strains except *Pseudomonas syringae* pv. *maculicola*. The 9 amino acids long C-terminal sequence (NCR147_28-36_) had activity against *Escherichia coli*, *Acinetobacter baumannii*, and the plant pathogenic *Pseudomonas syringae* strains and *Xanthomonas citri* pv. *malvacearum*. Replacement of tryptophan with 5-fluoro-L-tryptophan greatly broadened the antimicrobial spectrum and increased the activity resulting in 3.13 μM MBC in the case of 8 bacterium species. The positive effect of the W/W^F^ replacement in the enhancement of antibacterial activity was also observed in the case of NCR169 (Howan et al., 2023). In addition to fast killing, the NCR147 derivatives were potent inhibitors of biofilm formation, particularly the W/W^F^ substituted derivatives that were as effective as cefepime. Even more importantly, they were able to disperse existing biofilms that could not be achieved with the tested antibiotics.

Although *P. aeruginosa* and *P. syringae* are closely related species, their distinct lifestyles result in significantly different sensitivities to antimicrobial agents. *P. syringae* comprises at least 50 pathovars (Sohn et al., 2012) and can cause various types of plant diseases. It can colonize multiple plant tissues, including seeds, leaves, fruits, and bark. We examined three of them and found that they showed similar antimicrobial sensitivity to each other but differed significantly from the other pathogens. The *P. aeruginosa* which is an opportunistic human pathogen, most commonly found in hospital settings, where high exposure to antibiotics and disinfectants imposes strong selective pressure for the development of diverse resistance mechanisms. It employs multiple efflux pumps (e.g., MexAB-OprM, AmrAB-OprA, PmpM, MefA, ErmEPAF) to efficiently expel antimicrobial agents from the cell, produces numerous enzymes capable of degrading these compounds, and exhibits effective biofilm formation. Additionally, its outer membrane is less permeable to antimicrobial substances (Meletis et al., 2013; Verdial et al., 2023). In contrast, *P. syringae* strains, as plant pathogens in agricultural environments, are exposed to much lower antibiotic selective pressure. As a result, they possess less developed efflux systems, are less efficient in biofilm formation compared to *P. aeruginosa*, and overall face weaker evolutionary pressure to acquire resistance genes. These findings from the literature appear to support our results, which show that these plant pathogens became the most sensitive strains in our studies.

Tryptophan plays a role in interaction with bacterial membranes (Khemaissa et al., 2022) and the essential importance of the WAWK sequence suggested an interaction with membrane lipids. Indeed, NCR147_25-36_ showed binding to several lipids, most strongly to cardiolipin. Cardiolipin is a distinctive phospholipid predominantly found in bacterial membranes, especially in Gram-negative bacteria. It plays a critical role in maintaining membrane structure, supporting protein function, and enabling physiological adaptations (Gold et al., 2010; Wood, 2018; Yeo et al., 2023; Wang et al., 2024). Cardiolipin is a unique dimeric phospholipid with two, highly negatively charged phosphate headgroups making hydrogen bonding and electrostatic interactions key to its binding with proteins. Accordingly, among the proteines identified in the pull-down assay, several are directly or indirectly linked to cardiolipin-related functions such as CydA (Borisov et al., 2021), SecY (Corey et al., 2018), TatA (Rathmann et al., 2017) and lon (Minami et al., 2011).

Unlike NCR147_25-36_, W/W^F^ replacement in NCR147_25-36_ did not show binding to cardiolipin. However, we cannot rule out that the fluorine atom in W^F^ interferes with the activity of horseradish peroxidase used for detection of lipid bindings (Sliskovic et al., 2009). This assumption is also supported by the presence of proteins with cardiolipin-associated functions among the common hits of NCR147_25-36_ and NCR147_25-36_ W_33,35_/W^F^ (SecA, SecD, OmpA, AtpA and NuoC) with relatively high peptide counts. Moreover, testing only a few lipids on the commercial lipid strips, the interaction of NCR147_25-36_ W_33,35_/W^F^ with other bacterial membrane components cannot be excluded.

Both peptides and particularly NCR147_25-36_ W_33,35_/W^F^ inhibited the bacterial efflux pumps, evidenced by increased ethidium bromide accumulation in bacteria. Efflux pumps are bacterial transport proteins, which play a critical role in extruding intracellular substrates, including antibiotics, into the external environment, thereby contributing to antibiotic resistance (Thakur et al., 2021). In addition, both peptides induced rapid membrane permeabilization in *E. coli* and *A. baumannii* resulting in increased fluorescence of intracellular propidium iodide in line with their rapid killing action. These data demonstrate that NCR147 derivatives can inhibit bacterial efflux pumps while simultaneously permeabilizing the membrane for PI. These combined actions make these peptides particularly potent against bacteria, especially those with robust efflux-mediated antibiotic resistance. Cardiolipin, located in the inner membrane, plays a crucial structural role in the proper embedding and function of efflux pumps and other transmembrane proteins (Du et al., 2020). If the binding of NCR147 peptides alters this structural organization, it may simultaneously affect the efflux pump itself, the biofilm quality regulated by the pump, and antimicrobial resistance.

Knowing that NCRs can enter the bacterial cytosol, we studied the interaction of the NCR147_25-36_ and NCR147_25-36_ W_33,35_/W^F^ with *E. coli* proteins by isolating StrepII-tagged NCR147_25-36_ and NCR147_25-36_ W_33,35_/W^F^-protein complexes with affinity chromatography and identified the bound *E. coli* proteins with LC–MS/MS. Both peptides exhibited strong interactions with proteins involved in transcription and translation, including DNA-directed RNA polymerase subunits *β*, β’ and *α*, elongation factor Tu, translation initiation factor IF-2, and the 30S ribosomal protein S1, necessary for translation initiation and mRNA binding. Several additional ribosomal proteins were also identified; however, it remains unclear how many of them interact directly with the peptides, since the crude protein extract contains all ribosomal components and secondary interactions cannot be ruled out, therefore the binding of one or a few ribosomal proteins may facilitate the recruitment of their interacting partners.

Another group of prominent binding partners included the E2 (dihydrolipoyllysine residue acetyltransferase) and E3 (dihydrolipoyl dehydrogenase) components of the pyruvate dehydrogenase complex, as well as the dihydrolipoyllysine residue succinyltransferase component of the 2-oxoglutarate dehydrogenase complex, all of which share the dihydrolipoyl moiety. The E1 component of the pyruvate dehydrogenase complex was also identified, albeit with lower peptide counts, suggesting that binding of the E2 and E3 components might lead to the association of the entire complex. The pyruvate dehydrogenase complex serves a pivotal role in cellular metabolism by bridging glycolysis and the tricarboxylic acid (TCA) cycle. This enzyme complex catalyzes the oxidative decarboxylation of pyruvate to acetyl-CoA, a crucial metabolic intermediate. Targeting this complex could disrupt central metabolic processes, potentially perturbing energy production and biosynthetic pathways. Such an action would add another dimension to the antimicrobial mechanism of the peptides, amplifying their effectiveness against bacterial pathogens.

Additionally, outer membrane proteins (OmpA, OmpC, OmpF), chaperones (DnaK, GroEL, ProQ), and cell division proteins (DamX, FtsZ, MreB) were identified, though with lower peptide counts. Unique proteins identified exclusively in either the NCR147_25-36_ or NCR147_25-36_ W_33,35_/W^F^ complexes may point to functional differences between the W- and W^F^-containing peptides.

Given its broader antimicrobial spectrum, NCR147_25-36_ W_33,35_/W^F^ was further evaluated for antifungal activity against two clinically relevant fungal pathogens, *Candida albicans* and *Cryptococcus neoformans*. Both species were sensitive to the peptide, with minimum inhibitory concentrations (MICs) of 12.5 μM for *C. albicans* and 3.1 μM for *Cr. neoformans*. Importantly, NCR147_25-36_ W_33,35_/W^F^ did not induce hemolysis of human red blood cells, nor did it cause cytotoxicity or apoptosis in normal, non-cancerous human cells. These properties support its potential as a safe and selective therapeutic agent.

## 5 Conclusion

In conclusion, this study successfully identified shortened derivatives of the 36-amino-acid NCR147 peptide with potent antimicrobial activity against a broad spectrum of human and plant pathogens. While some derivatives exhibited narrow-spectrum activity, others were broadly effective. Notably, the 12-amino-acid variant NCR147_25–36_ W_33,35_/W^F^ demonstrated rapid and efficient bactericidal effects against several clinically relevant ESKAPE pathogens—including *Escherichia coli*, *Salmonella enterica*, *Acinetobacter baumannii*, *Pseudomonas aeruginosa*—as well as *Listeria monocytogenes*, *Staphylococcus aureus* and multiple plant pathogens. In addition to its antimicrobial potency, NCR147_25–36_ W_33,35_/W^F^ exhibited significant antibiofilm activity. Moreover, it was also effective against clinically relevant fungal pathogens.

Mechanistically, the peptide employs a multitarget mode of action, disrupting bacterial membranes and interfering with intracellular functions through interactions with various proteins, thereby reducing the likelihood of resistance development. Its cost-effective synthesis and absence of cytotoxicity on human cells further underscore its promise as a safe and practical antimicrobial candidate. Overall, this work underscores the potential of plant-derived peptides as an underexplored yet valuable resource for developing novel antimicrobial agents.

## Data Availability

The datasets presented in this study can be found in online repositories. The names of the repository/repositories and accession number(s) can be found in the article/Supplementary material.

## Footnotes

1
https://protifi.com/pages/protocols

2
https://dbaasp.org/toolspage=property-calculation

3
https://peptidechemistry.org/peptalyzer/
