# Supplementary material for: Multifaceted antimicrobial mechanisms of NCR147-derived peptides from Medicago truncatula
Source: Front Microbiol. 2026 Jan 27;16:1720738. doi: 10.3389/fmicb.2025.1720738 (PMC12886489; doi:10.3389/fmicb.2025.1720738)
Supplement: Supplementary file 2 [file Image_1.pdf]

NCR 147

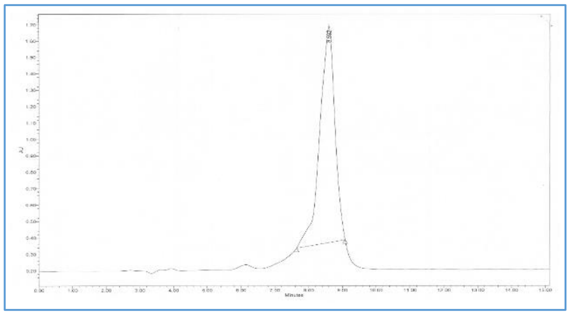

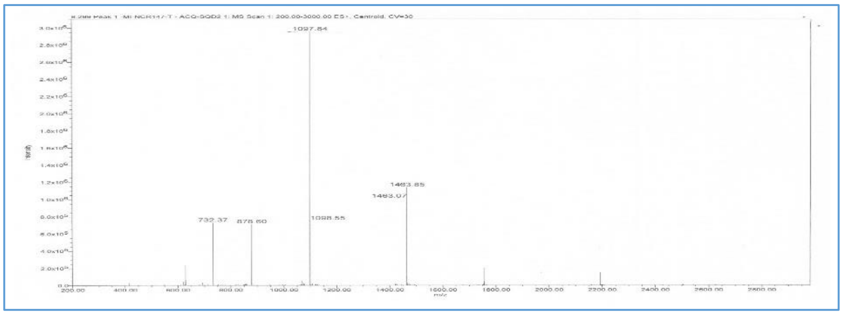

NCR147$_{13\text{-}36}$

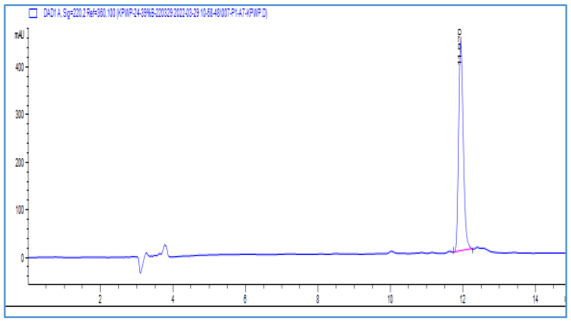

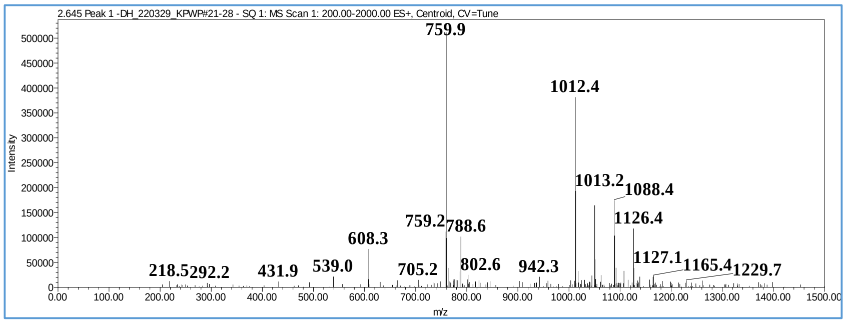

NCR147$_{25\text{-}36}$

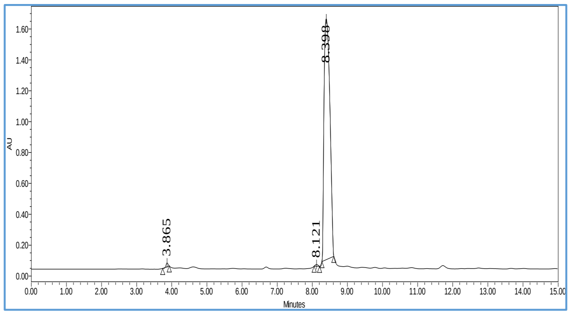

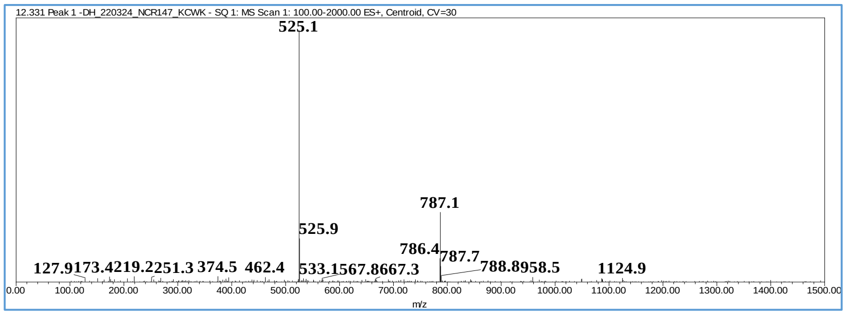

NCR147$_{25\text{-}36}$ox

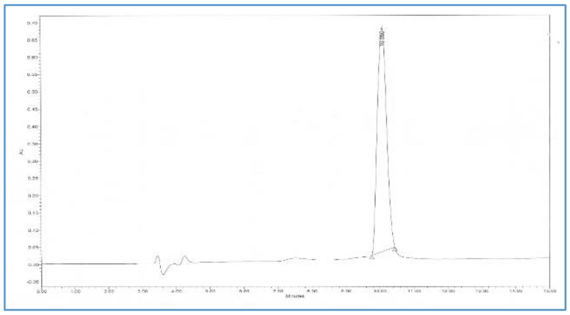

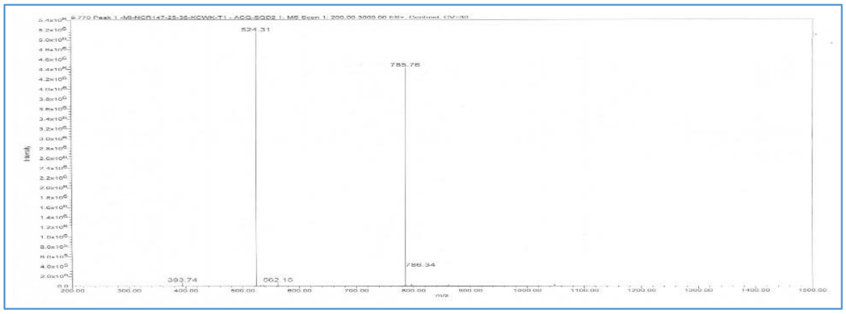

NCR147$_{20\text{-}36}$

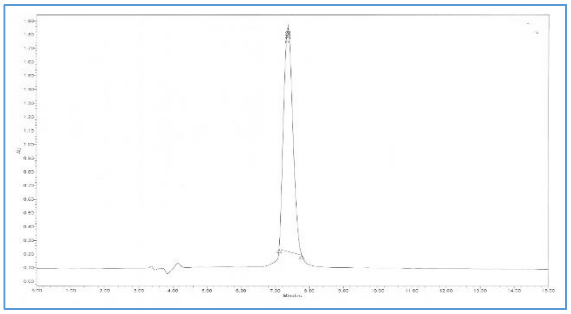

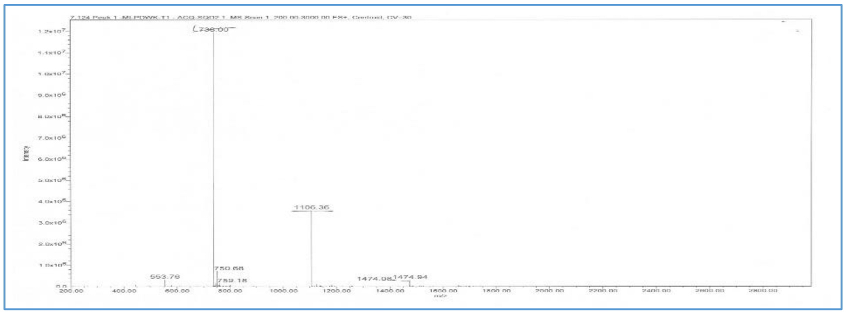

NCR147$_{25\text{-}36}$C$_{26,31}$/S

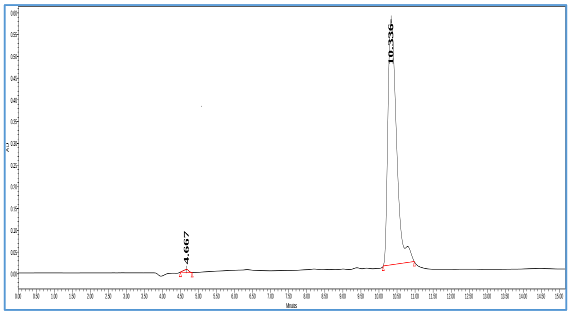

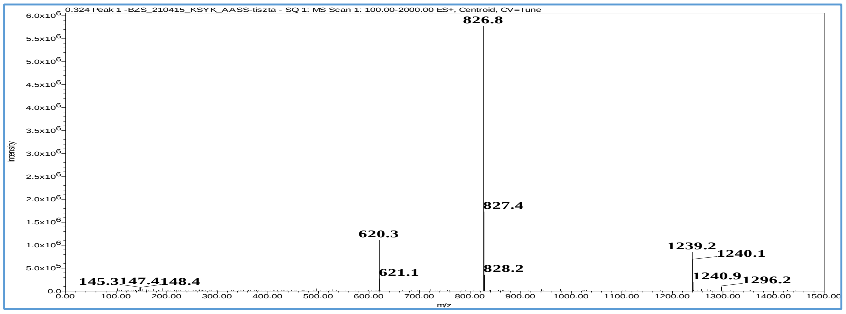

NCR147$_{25\text{-}32}$

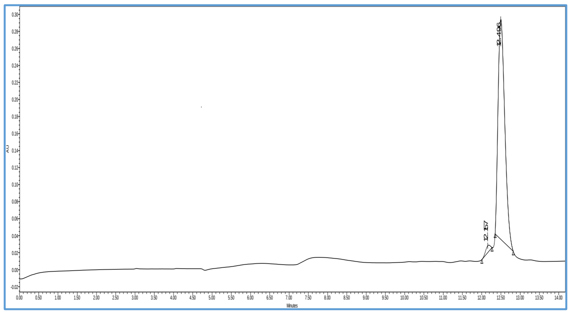

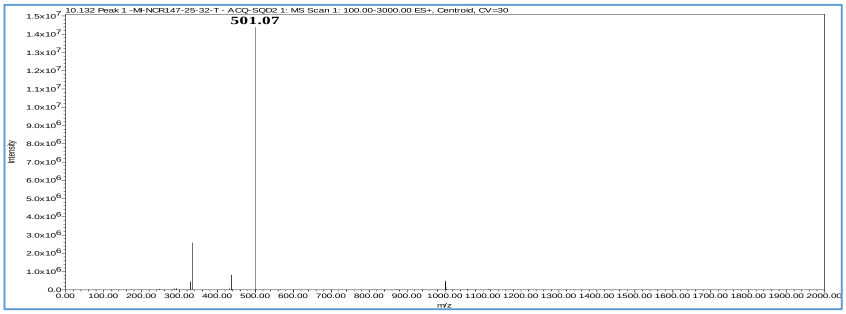

NCR147$_{25\text{-}36}$W$_{33}$/A

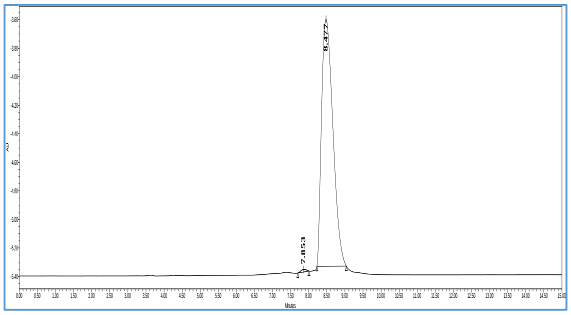

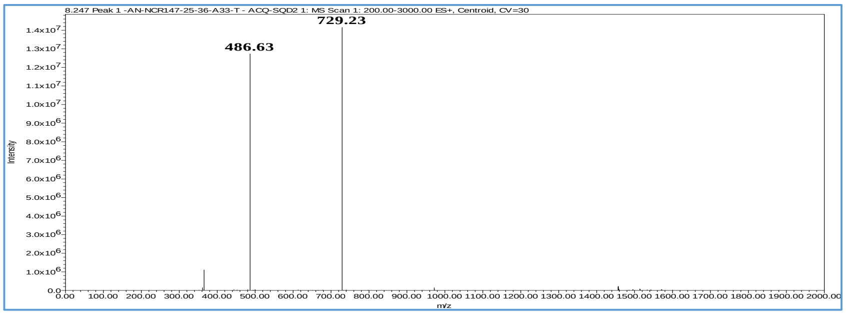

NCR147$_{25\text{-}36}$W$_{35}$/A

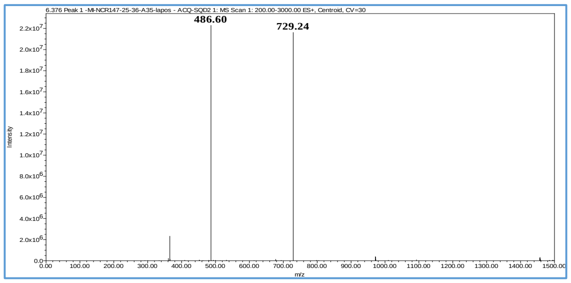

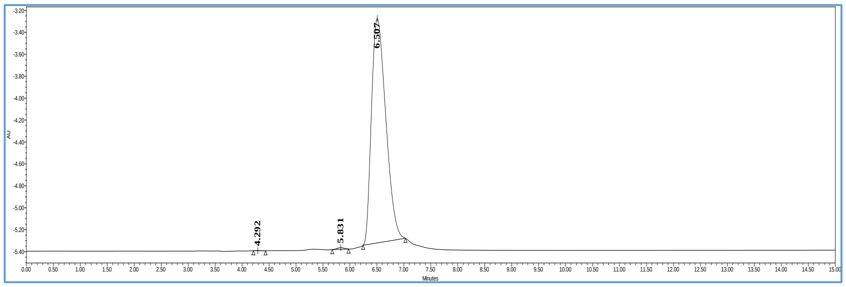

NCR147$_{25\text{-}36}$W$_{33,35}$/A

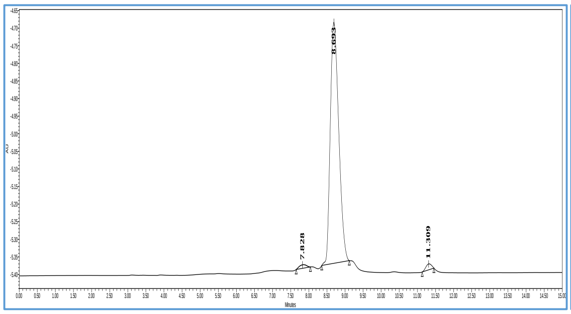

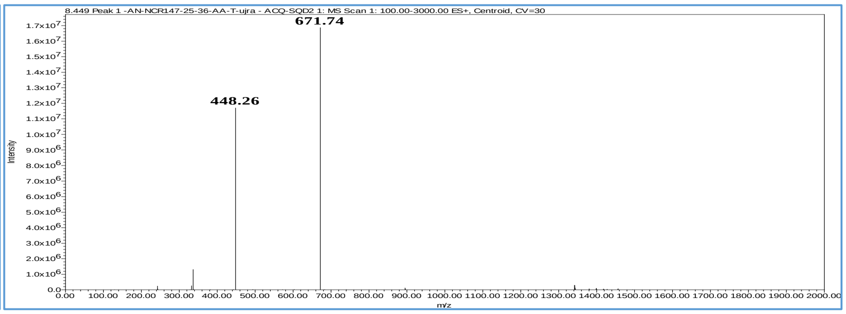

NCR147$_{25-36}$W$_{33}$/W^{F}

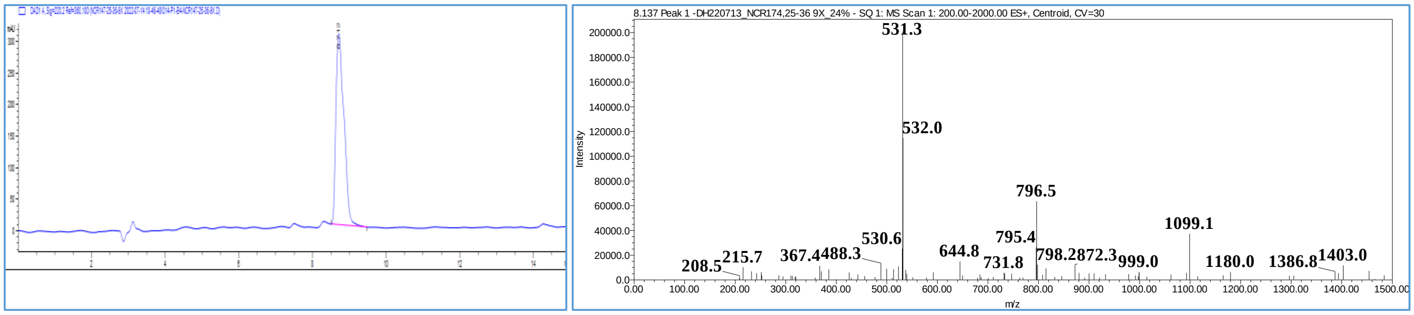

NCR147$_{25-36}$W$_{35}$/W^{F}

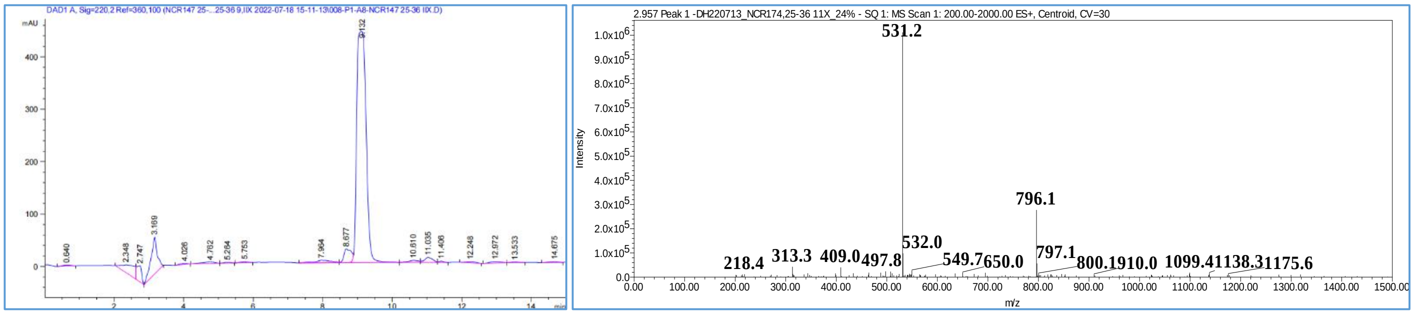

NCR147$_{25-36}$W$_{33,35}$/W^{F}

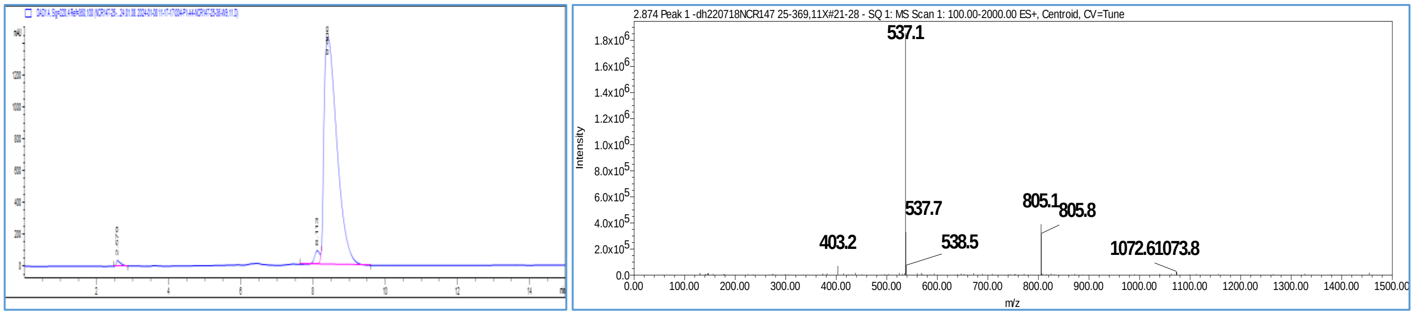

NCR147$_{28-36}$

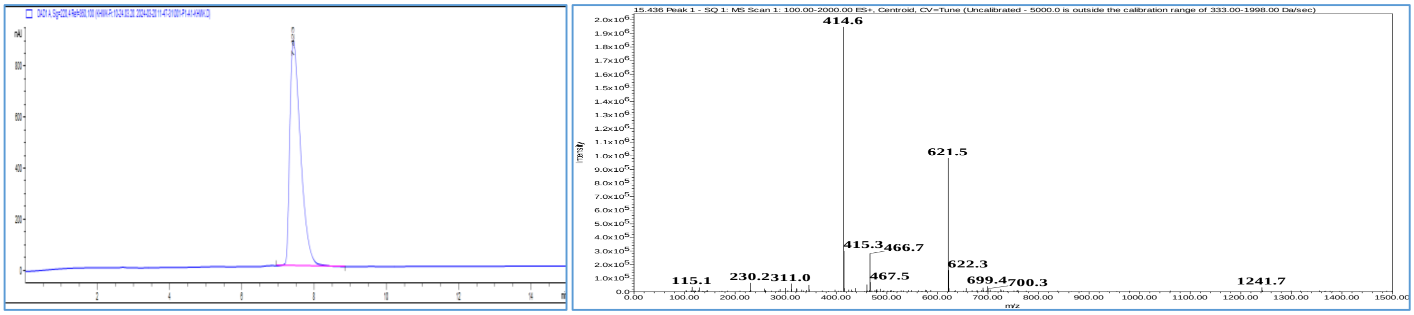

Supplementary Figure S1. **Purity profile of synthesized NCR peptides.** The HPLC profiles of the chemically synthesized peptides are shown in the left panels, while their electrospray ionisation mass spectra are shown in the right panels. The measured m/z values were in good agreement with the calculated ones. The purity of the peptides used was above 95% in all cases.
