# Supplementary material for: Multifaceted antimicrobial mechanisms of NCR147-derived peptides from Medicago truncatula
Source: Front Microbiol. 2026 Jan 27;16:1720738. doi: 10.3389/fmicb.2025.1720738 (PMC12886489; doi:10.3389/fmicb.2025.1720738)
Supplement: Supplementary file 3 [file Image_2.pdf]

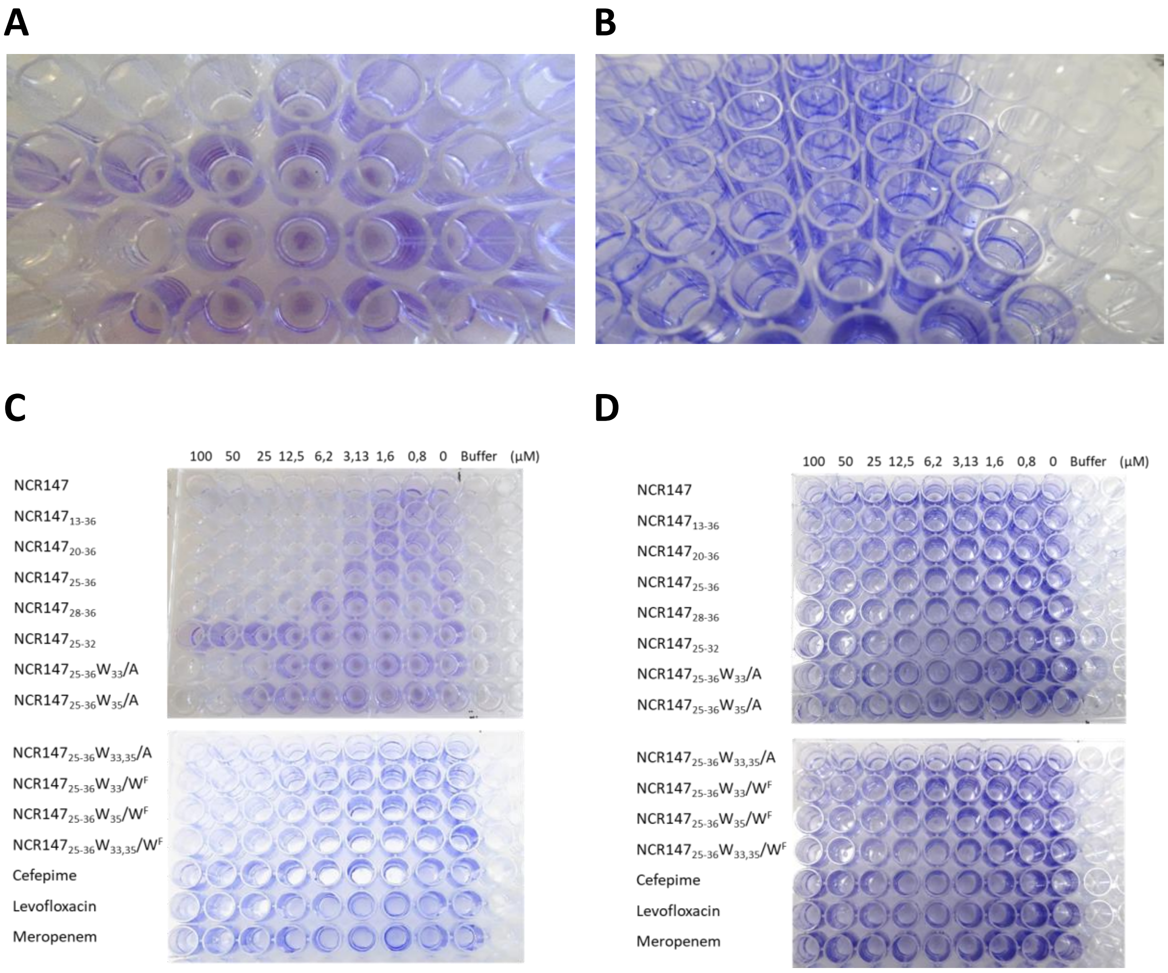

Supplementary Figure S2. **Formation, inhibition and eradication of *A. baumannii* biofilms in microtiter plates.** Biofilms of *A. baumannii* formed on the bottom of the wells (A) and along the air–liquid interface on the side walls of the wells in a characteristic ring-shaped pattern (B). Representative examples of biofilm inhibition (C) and eradication (D) following peptide and antibiotic treatments.
