# Supplementary material for: Multifaceted antimicrobial mechanisms of NCR147-derived peptides from Medicago truncatula
Source: Front Microbiol. 2026 Jan 27;16:1720738. doi: 10.3389/fmicb.2025.1720738 (PMC12886489; doi:10.3389/fmicb.2025.1720738)
Supplement: Supplementary file 4 [file Image_3.pdf]

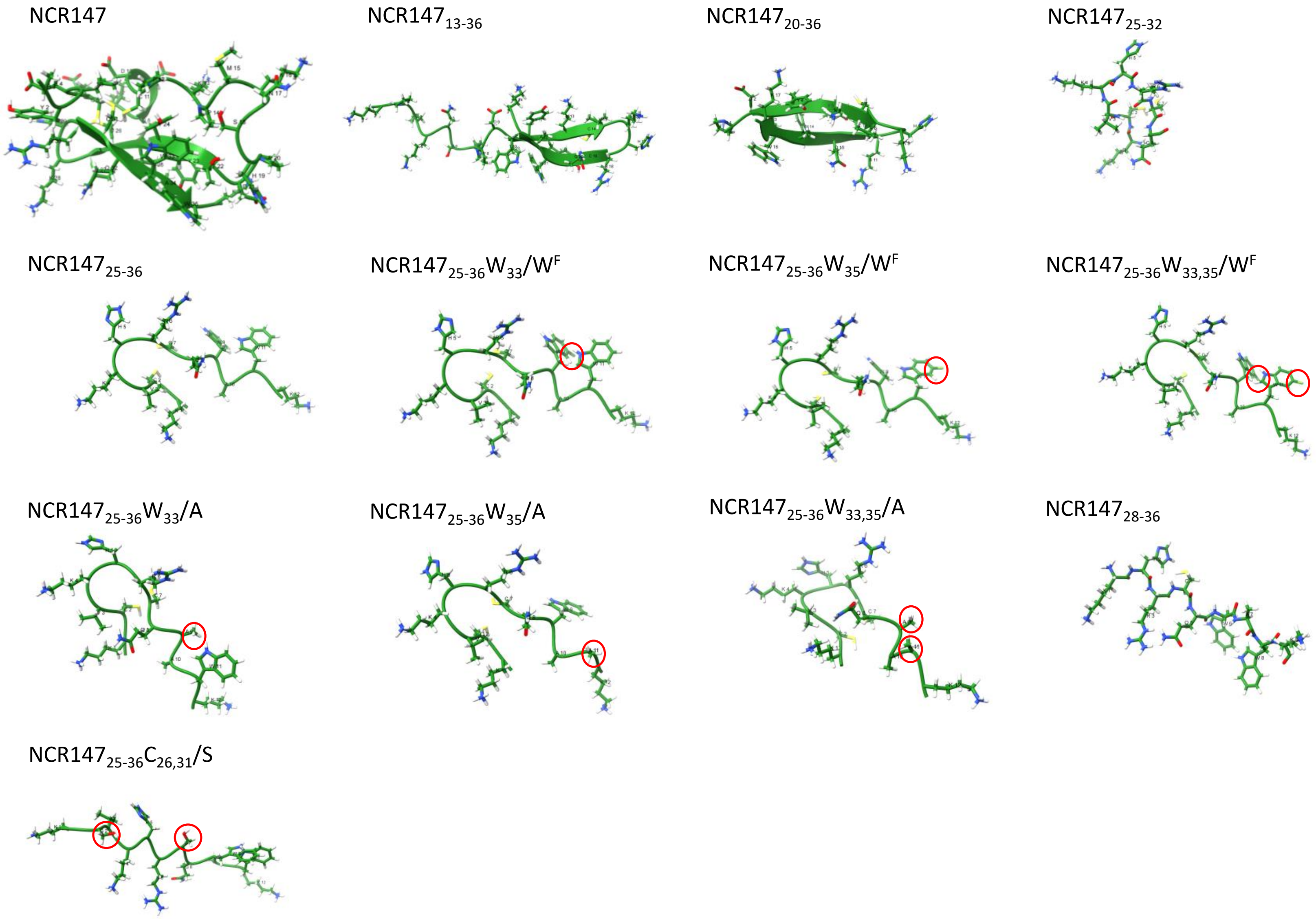

Supplementary Figure S3. Three-dimensional structural models of the NCR147 peptide and its derivatives. Structural predictions were generated using CSF ChimeraX version 1.9 (2024-12-11) in combination with ColabFold.
